# Stability of Nanopeptides: Structure and Molecular Exchange of Self-assembled Peptide Fibers

**DOI:** 10.1021/acsnano.3c01811

**Published:** 2023-06-26

**Authors:** Nico König, Szymon Mikolaj Szostak, Josefine Eilsø Nielsen, Martha Dunbar, Su Yang, Weike Chen, Ari Benjamin, Aurel Radulescu, Najet Mahmoudi, Lutz Willner, Sinan Keten, He Dong, Reidar Lund

**Affiliations:** † Department of Chemistry, University of Oslo, P.O. Box 1033 Blindern, 0315 Oslo, Norway; ‡ Jülich Centre for Neutron Science (JCNS-1) and Institute for Biological Information Processing (IBI-8), Forschungszentrum Jülich GmbH, 52425 Jülich, Germany; § Department of Mechanical Engineering, Northwestern University, Evanston, Illinois 60208, United States; ⊥ Department of Chemistry & Biochemistry, The University of Texas at Arlington, Arlington, Texas 76019, United States; ¶ Jülich Centre for Neutron Science (JCNS) at Heinz Maier-Leibnitz Zentrum (MLZ), Forschungszentrum Jülich GmbH, 85747 Garching, Germany; # ISIS-STFC, Rutherford Appleton Laboratory, Chilton, Oxon OX11 0QX, United Kingdom; ∥ Department of Civil and Environmental Engineering, Northwestern University, Evanston, Illinois 60208, United States; △ Hylleraas Centre for Quantum Molecular Sciences, University of Oslo, 0315 Oslo, Norway

**Keywords:** Peptide-assembly, nanostructured peptides, molecular exchange, structural stability, peptide−polymer conjugates, small-angle scattering, computer simulation

## Abstract

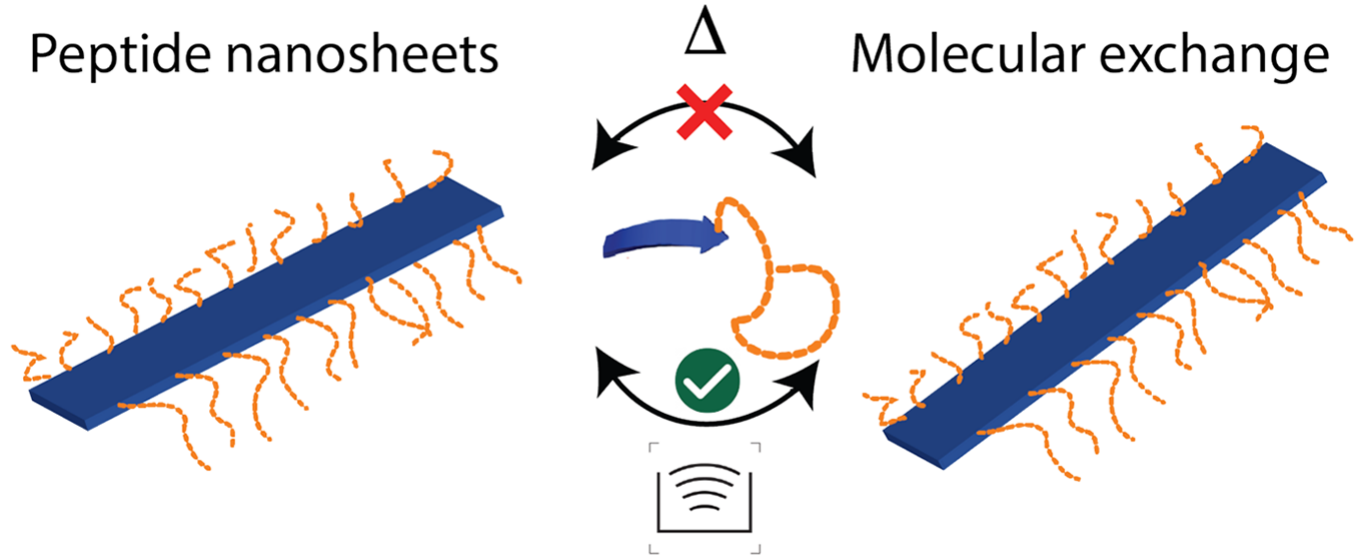

Often nanostructures formed by self-assembly of small molecules based on hydrophobic interactions are rather unstable, causing morphological changes or even dissolution when exposed to changes in aqueous media. In contrast, peptides offer precise control of the nanostructure through a range of molecular interactions where physical stability can be engineered in and, to a certain extent, decoupled from size via rational design. Here, we investigate a family of peptides that form beta-sheet nanofibers and demonstrate a remarkable physical stability even after attachment of poly(ethylene glycol). We employed small-angle neutron/X-ray scattering, circular dichroism spectroscopy, and molecular dynamics simulation techniques to investigate the detailed nanostructure, stability, and molecular exchange. The results for the most stable sequence did not reveal any structural alterations or unimer exchange for temperatures up to 85 °C in the biologically relevant pH range. Only under severe mechanical perturbation (i.e., tip sonication) would the fibers break up, which is reflected in a very high activation barrier for unimer exchange of ∼320 kJ/mol extracted from simulations. The results give important insight into the relation between molecular structure and stability of peptide nanostructure that is important for, e.g., biomedical applications.

## 1 Introduction

Self-assembly, that is, the autonomous association of building blocks into higher order structures, is a ubiquitous phenomenon found on all length scales, from nanostructures over living organisms to macroscopic processes like in weather systems.^1^ In the context of molecular self-assembly, it usually involves the noncovalent association of molecules into superstructures through free energy minimization,^2^ driven by various forces, among others hydrophobic and electrostatic interaction, van-der-Waals forces, or hydrogen bonds.^3^ Examples for molecular self-assembly range from systems of fundamental scientific or industrial interest like block copolymers and surfactants to biologically or medically relevant systems like lipid membranes, peptides, and proteins.^4^ While polymer assembly usually involves hydrophobically or electrostatically driven assembly of whole blocks, peptide and protein assembly is much more complex, involving defined sequences and motifs driving specific folding into a variety of nanostructures. Although molecular self-assembly has been studied for decades, there is still a lack of understanding of their nanostructure and their dynamic properties. As an example, even simple dipeptides assemble into a variety of nanostructures that cannot easily be predicted and require multiscale simulations and machine learning.^5,6^ For larger peptides, proteins, and hybrid systems, e.g., peptide–polymer conjugates, there is thus a considerable challenge to predict the structure and stability required to rationally design materials for, e.g., biomedical applications.

Extraordinarily stable proteins can be found in nature, for example, inside thermophilic bacteria (e.g., *Bacillus stearothermophilus*, *Thermus aquaticus*).^7^ A detailed comparison between amino acid compositions of enzymes present in mesophilic bacteria and analogous ones in thermophilic bacteria gives no universal answer to the cause of the extraordinary stability, but some trends are revealed including abundance of hydrophobic amino acids when compared to analogous, less thermally stable enzymes.^8^ Studies show that the origin of extraordinary stability in proteins comes from balancing hydrophobic interactions, hydrogen bonding, and electrostatic interactions.^9,10^ However, the structural stability of peptides may also pose severe challenges. For example, protein (mis)folding and assembly into more thermodynamically stable conformations underlie certain neurodegenerative diseases, such as Huntington’s, Alzheimer’s, Parkinson’s, or Creutzfeldt-Jakob, and are also associated with certain cancers.^11,12^ A prominent example is Alzheimer’s disease where proteins misfold into beta-sheets, which then self-assemble into protofilaments, subsequently fibrilize, and pack into amyloid fibers.^11,13^ Previous studies of laboratory derived amyloid nanofibers revealed the presence of π–π aromatic interactions between phenylalanine rings from neighboring molecules and salt-bridges between charge pairs (glutamic acid–lysine) in addition to hydrophobic interactions.^14^ The complexity of the process as well as lack of sequence specificity in β-amyloid peptides suggests involvement and balance of various intramolecular interactions, leading to extraordinary stability of nanofilaments.^15^ The fibrils associated with Alzheimer’s are so stable that the process leading to the formation of such “plaque” that in turn leads to synaptic damage and neurodegeneration is considered essentially irreversible.^16^ Hence, in order to understand the molecular basis of these diseases and guide possible treatments, it is essential to understand the thermodynamical and kinetic stability of such protein fibers and how they can be manipulated.^17^

Self-assembling peptides have also emerged as an important family of materials because of their diverse biological function and biocompatibility and are thus being extensively exploited for various biomedical applications such as drug delivery, gene delivery, vaccination, antimicrobial therapy, and regenerative medicine.^18−32^ In these applications, control over peptide nanostructures, as well as their dynamic stability, is of utmost importance to tune and optimize their biological activity and function. In some cases, where peptide unimers are functionalized with targeting ligands, therapeutic drugs, or imaging agents, disassembly exposes peptide unimers for rapid proteolytic degradation, leading to the excretion of therapeutics or imaging agents before reaching the relevant site for the disease. Thus, besides the chemical stability of the peptides themselves against proteolytic degradation, the physical stability of the assembled structures is of crucial importance for the development of self-assembled peptides for practical applications. While research on therapeutic peptide self-assembly is emerging, the study of their kinetic stability has not yet been explored to much extent compared to those for polymer-based delivery systems.^33−35^ A few groups, however, took steps forward to study the dynamic stability of peptide self-assembly and its effect on biological activity and function. For example, Stupp and co-workers showed that the cohesive force used to stabilize peptide amphiphile assembly plays an important role in mediating their cellular interactions and therefore cytotoxicity.^36^ Peptide assembly with stronger cohesion led to cell survival rates higher than those with weaker cohesion. Xu and co-workers reported on a class of amphiphilic peptide–poly(ethylene glycol) (PEG) conjugates that form core–shell spherical micelles.^37,38^ The kinetic stability of the micelles was significantly enhanced by forming an α-helical bundle in the headgroup, which slowed down the molecular exchange of the amphiphilic subunits in the micelle. Da Silva et al. observed that for long cylindrical peptide assemblies, stabilized by beta-sheet formation, the molecular exchange takes place on the time scale of hours and concluded that the exchange mechanism involved transfer of both unimeric molecules and small clusters.^39^ As shown by these examples, the internal dynamics, which are primarily governed by the interaction mediating the assembly,^40^ are highly important because they strongly influence the stability of the assembly and therefore their therapeutic efficacy.

Among these various peptide assemblies, self-assembled high-aspect-ratio nanofibers based on beta-sheet formation have attracted considerable attention not only in relation to aforementioned neurodegenerative diseases but also as biomaterials for therapeutic delivery, vaccination, and antimicrobial therapy.^19−21,23,26,30,41−44^ For example, Schneider and Pochan designed a family of beta-hairpin peptides that can self-assemble to form nanofibrous hydrogels.^45−48^ These peptide hydrogels have intrinsic antimicrobial activity and have been actively used as 3-D tissue scaffolds. Collier and co-workers developed an exquisite material platform using self-assembled beta-sheet nanofibers where multivalent vaccine epitopes are presented on the surface of these nanofibers, which improved vaccination efficacy.^49−52^ Nilsson and co-workers synthesized a library of self-assembling peptide nanofibers consisting of non-natural aromatic amino acids.^53−56^ The effect of aromatic interactions on the stability of the self-assembled peptide nanofibers was systematically studied, and these nanofibers are currently investigated for targeted therapeutic delivery.^57^ Furthermore, multidomain peptides developed by Hartgerink and Dong represent a family of molecular building blocks for the construction of peptide hydrogels as extracellular matrix mimetics as well as nanocarriers for anticancer and antimicrobial drug and gene delivery.^30,58−64^

While these peptide nanofibers show great promise toward practical biomedical applications, fundamental studies of their supramolecular structures and structure-dependent physical stability have rarely been performed.^3,4,17^ This is largely due to the challenge for the determination of dynamic exchange using conventional techniques such as fluorescence or temperature-jump experiments, as they require significant perturbation, either physically or chemically.

“PEGylation”, i.e., covalent attachment of poly(ethylene glycol) (PEG), also called poly(ethylene oxide) (PEO)), is an effective way to improve the solubility and increase the lifetime of therapeutic proteins and peptides in the blood stream.^65−68^ This is a result of shielding the epitopes of the peptide or protein leading to more difficult recognition by the immune system or simply an increase in the size of the conjugate, thereby inhibiting renal filtration and altering the biodistribution. However, PEG is also an unconventional polymer with amphiphilic character, which is reflected in a closed loop solubility behavior at high temperatures,^65,69^ good solubility in both aqueous and organic solvents, and significant activity at polar/nonpolar surfaces.^70^ In fact, it has recently been proposed that PEG itself behaves as a weak organic solvent^71^ and the solubility of PEG in aqueous solution is largely governed by its ability to hydrogen bond with other molecules.^69^ In biological systems, PEG may interact or solubilize certain amino acids.^71^ In previous work by Hamley et al.,^68,72^ Klok et al.,^73^ and Thiyagaran et al.,^74^ it was shown that PEGylation does not necessarily disturb the intrinsic structure of the peptide assemblies, but it may provide additional stability toward fibril aggregation and precipitations caused by, e.g., changes in pH.

In this work, we address the aforementioned challenges by employing small-angle X-ray/neutron scattering (SAXS/SANS) techniques, supported by differential scanning calorimetry (DSC) and circular dichroism (CD) spectroscopy, to investigate the physical stability of self-assembled multidomain peptides that can be regarded as a prototype of beta-sheet peptide nanofibers. We investigated peptides with the sequences K_3_W(QL)_6_K_2_, K_4_W(QL)_6_K_2_, and K_5_W(QL)_6_K_2_, which are based on the general K_*x*_(QL)_*y*_K_*z*_ multidomain peptide motif proposed by Dong et al.^58^ As a reference, we also examined the peptide W(QL)_3_K_5_(QL)_3_ as an example of an isomer of K_3_W(QL)_6_K_2_ without a long multidomain peptide motif. Using transmission electron microscopy (TEM), circular dichroism spectroscopy (CD), and small-angle X-ray scattering (SAXS), Yang et al. showed that these peptides self-assemble into elongated fibers consisting of two stacked beta sheets, held together by hydrophobic interaction of the leucine residues.^59^ The assembly is the result of a delicate balance between attractive (hydrogen bonds and hydrophobic interactions) and repulsive forces (electrostatic repulsion of terminal lysine residues at physiological pH). In addition, considering the needs for PEGylation on many nanofibers for practical biomedical applications, we also investigated multidomain peptide–PEG conjugates PEG-K_3_W(QL)_6_K_2_, PEG-K_4_W(QL)_6_K_2_, PEG-K_5_W(QL)_6_K_2_, and PEG-W(QL)_3_K_5_(QL)_3_. For the peptide PEG-K_3_W(QL)_6_K_2_, by isotopically labeling the polymer chain (deuterated or proteated) and using time-resolved SANS, we were able to assess the kinetic stability of the fibers. We found the multidomain peptide beta-sheet nanofibers to be exceptionally stable, withstanding all tested conditions without structural alterations (as resolvable by SAXS/SANS). We also found practically no thermally activated molecular exchange between the fibers. Only under strong mechanical agitation would molecular exchange take place via fiber break-up and reformation. The results show that robust nanostructures with little susceptibility toward perturbations can be engineered by using relatively short beta-sheet-forming peptides. These peptide assemblies have superior stability as compared to regular surfactant-like amphiphiles because of the combination of hydrophobic interactions between the leucine residues and the strong hydrogen bonding structure between glutamines. Our results highlight the nanostructural stability that can be achieved by peptide assembly and further be exploited for biomedical applications where the structural integrity is required to, e.g., prevent enzymatic degradation that otherwise more easily would take place for small, nonassembled (unimeric) peptides.^36−38^

## 2 Results and Discussion

In this work, we investigated peptides containing a multidomain peptide motif, with the general sequence K_*x*_(QL)_*y*_K_*z*_. We examined the stability of the nanofibers with increasing number of lysine residues (increasing the overall positive charge of the peptide and thereby also the electrostatic repulsion) and compared their stability with an isomer of K_3_W(QL)_6_K_2_ peptide with a disrupted (QL)_*y*_ motif. The presence of tryptophan residue allows for accurate peptide concentration determination while preserving the self-assembled structure.

We will first present the results we obtained from the non-PEGylated K_3_W(QL)_6_K_2_ peptide, then continue with insights we gained from the K_4_W(QL)_6_K_2_, K_5_W(QL)_6_K_2_, and W(QL)_3_K_5_(QL)_3_, followed by conclusions from analysis of PEGylated versions of peptides PEG-K_3_W(QL)_6_K_2_, PEG-K_4_W(QL)_6_K_2_, PEG-K_5_W(QL)_6_K_2_, PEG-W(QL)_3_K_5_(QL)_3_, and finally discuss the physical integrity of the peptide fibers based on time-resolved SANS experiments with a PEG-K_3_W(QL)_6_K_2_ + dPEGK_3_W(QL)_6_K_2_ mixture.

### 2.1 Peptide K_3_W(QL)_6_K_2_

**Figure 1:**
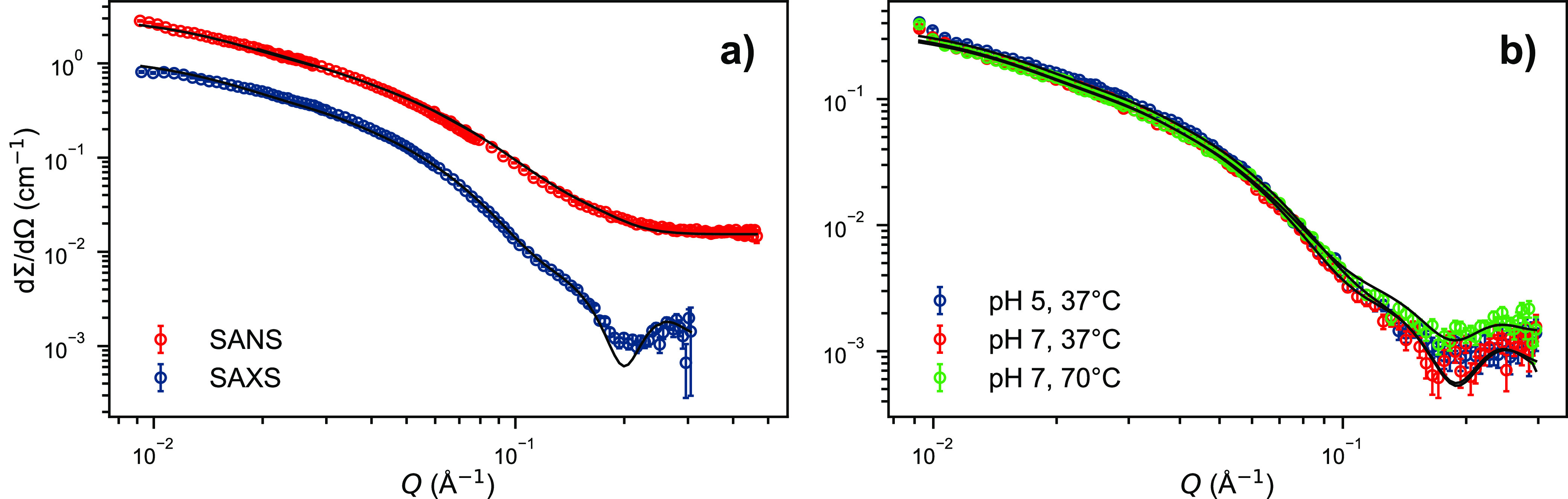
Small-angle scattering curves of K_3_W(QL)_6_K_2_, and black lines are model fits. a) The same 10 mg/mL peptide sample in pH 7.4 Tris buffer, measured with SAXS and SANS (100% D_2_O) at 25 °C. Both curves were fitted with the same set of parameters, see Table 1. b) SAXS curves of the K_3_W(QL)_6_K_2_ in pH 7.4 Tris buffer and pH 5.0 MES buffer, at 37 and 70 °C.

Both SAXS and SANS scattering curves from peptide K_3_W(QL)_6_K_2_ in D_2_O are presented in Figure 1, showing similar features with a *Q*^–2^ slope at low *Q*, where *Q* is defined as  and is inversely proportional to the measured correlation distance. The data at high *Q* thus provide information about the local structural features, while low *Q* reveals the overall morphology and size. The scattered intensity indicates the formation of elongated fiber-like structures. In addition, the oscillations observed at high *Q* are indicative of local structuring of the peptide chains. Preliminary analysis showed that a simple uniform filament-like scattering model, i.e., of a homogeneous sheet with dimensions,*a* < *b* < *c*, does not describe the scattering patterns at high *Q*. If we instead assume that the peptides assemble to form a sandwich-like structure, we obtain a much better fit. Here, a predominant *trans* conformation of the peptide strands provides a leucine- and glutamine-rich side, which associates via hydrogen bonds and hydrophobic interactions to form elongated nanosheets. This yields core–shell like nanosheets with an inhomogeneous electron density (lower density in the interior (core) than the outer part (shell)). To extract quantitative information for the dimensions of the nanostructure, we employed the core–shell model presented in the Experimental Section. As seen in Figure 1a, the model can jointly reproduce both neutron and X-ray scattering data on an absolute scale, yielding accurate structural parameters. The set of parameters of the simultaneous fits are provided in Table 1.

**Table 1:** Parameters of a Simultaneous Fit of SAXS and SANS^a^

*a*_core_ (Å)	9.8	*d*_core_ (g/mL)	0.95
*a*_shell_ (Å)	8.4	*d*_shell_ (g/mL)	1.36
*b*_core_ (Å)	44.1	*d*_pep_ (g/mL)	1.25
*b*_shell_ (Å)	8.5	ρ_core,*X*_ (cm^–2^)	9.07 × 10^10^
*c* (Å)	340	ρ_shell,*X*_ (cm^–2^)	1.23 × 10^11^
*N*_agg_	180	ρ_solv,*X*_ (cm^–2^)	9.37 × 10^10^
*M*_core_ (Da)	473	ρ_core,*N*_ (cm^–2^)	–3.77 × 10^8^
*M*_shell_ (Da)	1866	ρ_shell,*N*_ (cm^–2^)	4.06 × 10^10^
*M*_pep_ (Da)	2339	ρ_solv,*N*_ (cm^–2^)	6.35 × 10^10^

a Data are presented in Figure 1a.

The fit parameters are in good agreement with previously reported values, where a uniform filament model instead of core–shell model was used (*a*_pep_ = 26.6 Å vs 27 Å; *b*_pep_ = 61.1 Å vs 57 Å; *c* = 340 Å vs 400–500 Å).^60^

The self-assembly of K_3_W(QL)_6_K_2_ into fibers relies on a delicate balance between the hydrogen bonds caused by the glutamine units, hydrophobic interaction between the leucine residues, and the electrostatic repulsion between the terminal lysine residues.^59,60,75^ The fiber stability was further investigated at pH = 5, a relevant physiological pH for intracellular conditions. The peptide was dissolved in pH 5.0 MES buffer in an attempt to ionize the lysine residues and to thus enhance the repulsion. However, it can be observed in Figure 1b that the scattering pattern of the pH 5.0 sample is not different from that of a pH 7.4 sample. The reduction in pH does not seem to alter the overall fiber morphology, as resolvable by small-angle scattering. This is consistent with the circular dichroism (CD) measurements shown in Figure S6 in the Supporting Information (SI) where the secondary structure of K_3_W(QL)_6_K_2_ does not seem to change much upon pH reduction. In addition, we heated a peptide sample in Tris buffer (pH 7.4 at 25 °C) up to 70 °C. Due to the high temperature coefficient of Tris (d*pK*_*a*_/d*T* ≈ −0.03 K^–1^), this also means a significant reduction in pH (∼pH 6 @70 °C). Nevertheless, there still is no significant change in the scattering signal. The visible changes can be fully explained by the change of the solvent density with temperature and thus a change in the contrast conditions. The scattering data of all three curves presented in Figure 1b) can still be fitted with parameters very close to those used for the data in Figure 1a) which are given in Table 1. Thus, the peptide fibers exhibit great stability toward thermal perturbation up to 70 °C and reduction in pH. Regarding the interactions governing the assembly, this means that hydrophobic interaction (between the hydrophobic residues in the fiber core) and hydrogen bonds (between the peptide backbones within the beta-sheets) strongly dominate over electrostatic repulsion (terminal lysine residues), even if the latter is promoted via a lower pH. The net attractive forces are also able to withstand a significantly increased amount of thermal fluctuations. Although previously reported CD measurements revealed β-sheet melting temperatures of 67 and 65 °C for K_3_W(QL)_6_K_2_ and PEG-K_3_W(QL)_6_K_2_, respectively,^75^ we have not observed any signal in DSC measurements in these regions. Although the measurements were done in another buffer and at lower concentrations, the results indicate that although the conformation, e.g., the hydrogen-bond structure may be altered, the hydrophobic interactions maintain the integrity of the nanostructure. It might also be that the CD signal is sensitive to subtle changes in the twisting of the nanofibers that are not easily detectable in other methods.

### 2.2 Molecular Dynamics Simulations

**Figure 2:**
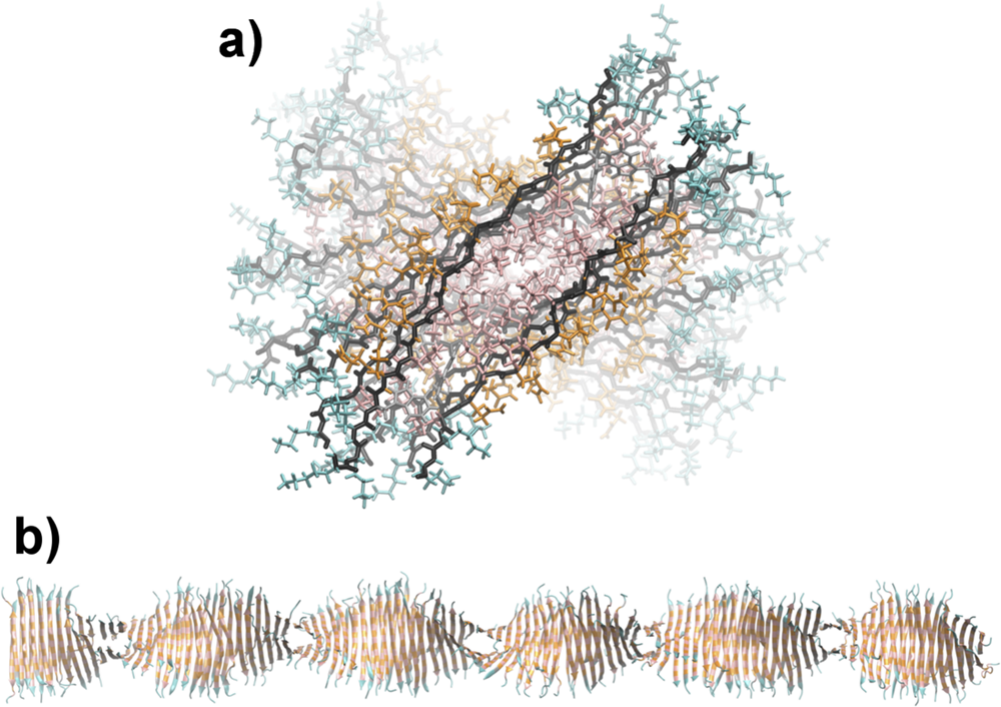
a) Cross-section view of the non-PEGylated K_3_W(QL)_6_K_2_ peptide fiber, where the peptide backbone is black, tryptophan is gray, lysine is teal, glutamine is orange, and leucine is pink. b) A representative snapshot of the whole peptide fiber after 15 ns equilibration.

The scattering experiments presented above yield only low-resolution information about the fiber structure. To shed more light on the internal packing of the peptide molecules, we performed molecular dynamics simulations on a 200-strand peptide assembly. As can be seen in Figure 2a, the geometrical scattering model of the peptide fiber agrees well with the simulated structure, which has a tightly packed hydrophobic core consisting of 6 leucine and 1 tryptophan residues (per strand). Additionally, the peptide assembly was determined to be extremely stable and composed of approximately 72% beta-sheet as measured by the STRIDE algorithm.^76^ This is in part due to the complete exclusion of hydrogen-bond-disrupting water molecules from the hydrophobic core, as no water penetrated further than the bulky tryptophan residue, even at the ends of the peptide exposed to solvent. Finally, MM-GBSA was used to determine the binding energy of the peptide. The binding energy is calculated to be 319.4(277) kJ/mol, which will later be shown to agree reasonably well with a rough empirical estimate of the binding energy. Additionally, to better understand the contribution of each residue in the peptide unimer (ligand) to the binding energy, the per-residue energy decomposition was calculated.^77^ As expected, the leucine residues in the ligand were found to contribute strongly to binding, with an average per-residue energy of (19.0 ± 2.7) kJ/mol, primarily through the van-der-Waals component. While glutamine was found to have larger electrostatic and polar solvation contributions than leucine, they tend to cancel out, and as a result, the van-der-Waals component is also the primary contributor to binding for glutamine, leading to an average per-residue energy of (12.8 ± 2.1) kJ/mol. Summing the anticorrelated electrostatic and polar solvation components of the lysine residue contribution indicated that the electrostatic repulsion is much larger than the polar solvation term. Although the repulsive contribution is tempered somewhat by the reasonably strong van-der-Waals interactions ((11.3 ± 6.2) kJ/mol), the overall energetic contribution from lysine is unfavorable to binding ((−11.5 ± 7.8) kJ/mol). In addition, we have to take into account the entropic contribution from the peptide residues. Among the cationic residues, lysine is known to provide additional stabilization of structured proteins because of its rather small size and flexibility leading to a significant configurational entropy (amount of rotamers) in the folded state,^78^ thereby stabilizing the structure.

Next, to confirm consistency with our experimental results, we calculated scattering curves from the simulated peptide fiber structure, shown in Figure 2b. Theoretical SAXS data were calculated with CRYSOL,^79^ and SANS data with CRYSON,^80^ using the program default parameters. We added a constant background to the simulated curves to account for insufficient background subtraction in the experimental data. As seen in Figure S8 in the SI, the curves concur favorably, strengthening the confidence in our MD simulation results. There is one obvious inconsistency, though: the MD peptide fiber is twisted, with a full-turn period of about 35 strands (∼165 Å), which might reduce the repulsion between the charged lysine residues. This feature is not reflected in the geometrical scattering model, which might be partly masked by the rotational average and the rather long periodicity. The other structural features, which are more locally defined, however, agree well with the fit parameters found for the geometrical scattering model (Table 1), with *a*_core_ = (13.3 ± 1.7) Å, *a*_shell_ = (5.2 ± 2.4) Å, *b*_core_ = (40.1 ± 0.6) Å, *b*_shell_ = (8.8 ± 1.2) Å, and *d*_pep_ ≈ 1.26 g/cm^3^.

### 2.3 Deconvoluting the Effect of Charge and Hydrophobicity

As is evident from the discussion so far, the QL-repeat provides significant structural stability through hydrogen bonding and hydrophobic interactions, which is partly counterbalanced by repulsive electrostatic interactions caused by charged lysine residues. In order to deconvolute the molecular interactions, we designed supplementary peptide sequences and performed additional experiments, where we systematically varied factors contributing to the stability of the sheets. Focusing on the electrostatic contributions first, we investigated how changing the number of lysine residues and thereby increasing the positive net charge at physiologically relevant pHs affects the integrity of the β-sheet motif and thus the resulting nanostructure. We specifically examined peptides K_4_(QL)_6_K_2_ and K_5_(QL)_6_K_2_, which have one and two additional lysines, respectively, and a “scrambled” isomer of K_3_(QL)_6_K_2_ where the lysine residues are moved to the middle of the QL repeating motif, W(QL)_3_K_5_(QL)_3_.

**Figure 3:**
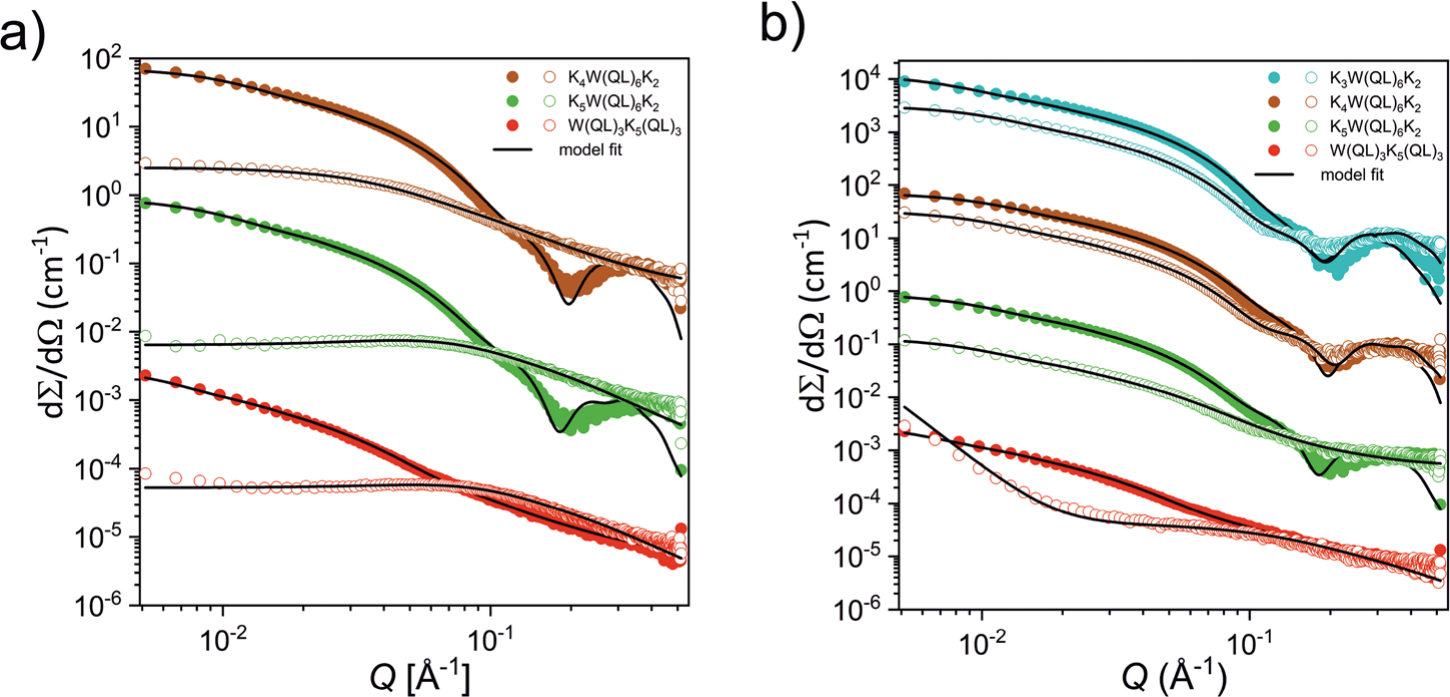
a) SAXS curves of the K_4_W(QL)_6_K_2_, K_5_W(QL)_6_K_2_, and W(QL)_3_K_5_(QL)_3_ peptides at the concentration 5 mg/mL in pH 7.4 Tris buffer (filled dots) and in pH 5 MES buffer (hollow dots); black lines represent model fits. To avoid overlapping, the intensity of K_4_W(QL)_6_K_2_ was multiplied by 100 and the intensity of W(QL)_3_K_5_(QL)_3_ by 0.01. b) SAXS curves of the K_3_W(QL)_6_K_2_, K_4_W(QL)_6_K_2_, K_5_W(QL)_6_K_2_, and W(QL)_3_K_5_(QL)_3_ peptides at the concentration 5 mg/mL in pH 7.4 Tris buffer (filled dots) and 5 mg/mL solutions of peptides containing 4 M urea (hollow dots); black lines represent model fit. To avoid overlapping, the intensity of K_3_W(QL)_6_K_2_ was multiplied by 10,000, the intensity of K_4_W(QL)_6_K_2_ by 100. and the intensity of W(QL)_3_K_5_(QL)_3_ by 0.01. In graphs, every third data point is displayed.

Figure 3a shows the SAXS data for the peptides at pH = 7.4 and 5. As seen, in contrast to the K_3_W(QL)_6_K_2_ peptide, the nanostructures of these peptides are significantly affected by changes to the pH. The scattering intensities of K_4_W(QL)_6_K_2_ and K_5_W(QL)_6_K_2_ both decrease drastically at pH = 5, as compared to the data at pH = 7.4, and a complete change in the overall shape of the scattering curve is observed. Model fit analysis of these data reveals that whereas K_4_W(QL)_6_K_2_ and K_5_W(QL)_6_K_2_ at pH = 7.4 are both well described by the core–shell nanosheet model, at pH = 5, the data are better described using the Beaucage scattering model for random (unaggregated) polymer chains.^81^ For K_5_W(QL)_6_K_2_, we also observe a clear maximum at intermediate *Q* indicative of strong electrostatic repulsion, which can be described by including a structure factor in the model.^82^ The resulting fits reveal a *M*_*w*_ of about 2200 g/mol and a radius of gyration, *R*_*g*_, of about 22 Å, which both are in excellent agreement with the assumption of completely dissolved peptide chains with residual repulsion caused by the high charge. For K_4_W(QL)_6_K_2_, we observe a larger molecular weight of about 8000 g/mol, which is about 4-times larger than a single peptide. This is consistent with a larger *R*_*g*_ of about 40 Å and the fact that the scattering curves do not show a structure factor peak, indicating that the peptides are not completely molecularly dispersed. Rather it seems that we have oligomeric aggregates that can be described only crudely using a simple scattering form factor for random polymer chain. Thus, the data clearly show that the additional lysine residues lead to a high effective charge at this pH, causing repulsions that are not sufficiently balanced by the hydrogen bonding structure of the glutamine units and peptide backbone. It is also likely that the repulsion weakens the H-bonds by perturbing the optimal alignment, thereby weakening the cohesive energy. Finally we note that the “scrambled” isomer of K_3_(QL)_6_K_2_, the W(QL)_3_K_5_(QL)_3_ peptide, with the same overall charge, is not able to form well-defined segregated nanosheets at any pH. This is not surprising as the QL repeating motif is interrupted by the lysine stretch, which inhibits both the hydrophobic association and effective hydrogen-bond formation. At pH = 5, the data show dominance of completely dissolved chains and some larger aggregates visible as a steep (∼*Q*^–4^) upturn at low *Q*.

In order to further understand the stability and driving force for nanosheet formation, we proceeded with examining the effect of urea, a well-known denaturant of proteins that acts by weakening the hydrophobic interactions.^83^ For purely hydrophobically driven self-assembled structures, we thus expect near complete dissolution with large amounts of urea.^84^Figure 3b shows the scattering patterns for various peptides in 50 mM Tris buffer and 4 M urea + 50 mM Tris buffer, where the overall contrast (difference in electron density) between the peptide and solvent diminishes with addition of urea, thereby reducing the overall intensity. Analysis of the K_3_W(QL)_6_K_2_ and K_4_W(QL)_6_K_2_ scattering patterns reveals that both show clear oscillation at high *Q* and typical scattering patterns of nanosheets also in 4 M urea. In fact, after taking into account the reduced electron density contrast, the overall scattering pattern and intensity can be described by using almost the same fit parameters for the K_3_W(QL)_6_K_2_ nanosheets in Tris buffer as well as in 4 M urea. We obtained essentially the same cross-section (about 2 × 5.7 nm), although the scattering at low *Q* indicated a slightly increased length. However, the exact length is not possible to be resolved since we do not observe a Guinier region and thus the length is still longer than the experimental *Q* resolution with this setup. For the K_4_W(QL)_6_K_2_ peptide, we found essentially the same scattering pattern. However, the structure of the peptide with the highest charge, K_5_W(QL)_6_K_2_, is changed by addition of urea. In scattering patterns of K_3_W(QL)_6_K_2_ and K_4_W(QL)_6_K_2_ peptides, the pronounced minimum/maximum (∼*Q* = 0.2 Å^–1^) characterize the well-segregated, relatively low electron density interior and electron-rich exterior of the sandwich-like core–shell nanosheet structure. Contrary to this, scattering data of K_5_W(QL)_6_K_2_ can be described by the model assuming more irregular sheetlike filaments that lack the sandwich-like segregated structures that were visible at pH = 7.4. The data clearly show that hydrogen bonds significantly contribute to the overall structural integrity of the nanofibers. Destabilization of the sheet structure is only observed for longer stretches of lysines where the addition of urea causes sufficient weakening of the hydrophobic interaction that cannot counterbalance the increased repulsions. The H-bonds thus contribute to a significant fraction of the binding energy, which, as shown by computer simulation, is very large for this system.

In order to analyze the thermal stability of K_4_W(QL)_6_K_2_, K_5_W(QL)_6_K_2_, and W(QL)_3_K_5_(QL)_3_ peptides, we performed a series of SAXS measurements in the temperature range 20–67 °C, which are presented in Figure S7 in the SI. We observed that the peptide K_4_W(QL)_6_K_2_ exhibits good thermal stability in the examined range, while the supramolecular structure of peptide K_5_W(QL)_6_K_2_ is slightly disrupted above 57 °C and that peptide W(QL)_3_K_5_(QL)_3_ does not self-assemble in that temperature range at all. The slight decrease in the total intensity is caused by a change of density of the solution resulting in a decrease in contrast. These results again indicate a very stable fiber structure.

Small angle scattering measurements allowed us to analyze the morphology of the nanofibers and their general shape and stability and gave insight about the electron density distribution within the structure. In order to better understand the internal structure, we performed an in-depth circular dichroism characterization of the fibers. All the spectra, together with model fits, are presented in Figure S6, and the determined distribution of secondary structure is summarized in Table S1. CD spectroscopy provides semiquantitative results about secondary structural components in nanofibers. However, additionally, the CD results might be sensitive to subtle changes in the twisting of the overall fiber-structure. It showed a reduction of the β sheet secondary structure as the number of lysine residues increased and pH of the solution decreased, suggesting the important role of electrostatic repulsion. This is consistent with SAXS results that indicate that additional lysine residues destabilize the packing and cause partial dissolution of the nanofibers.

### 2.4 Effect of PEGylation

**Figure 4:**
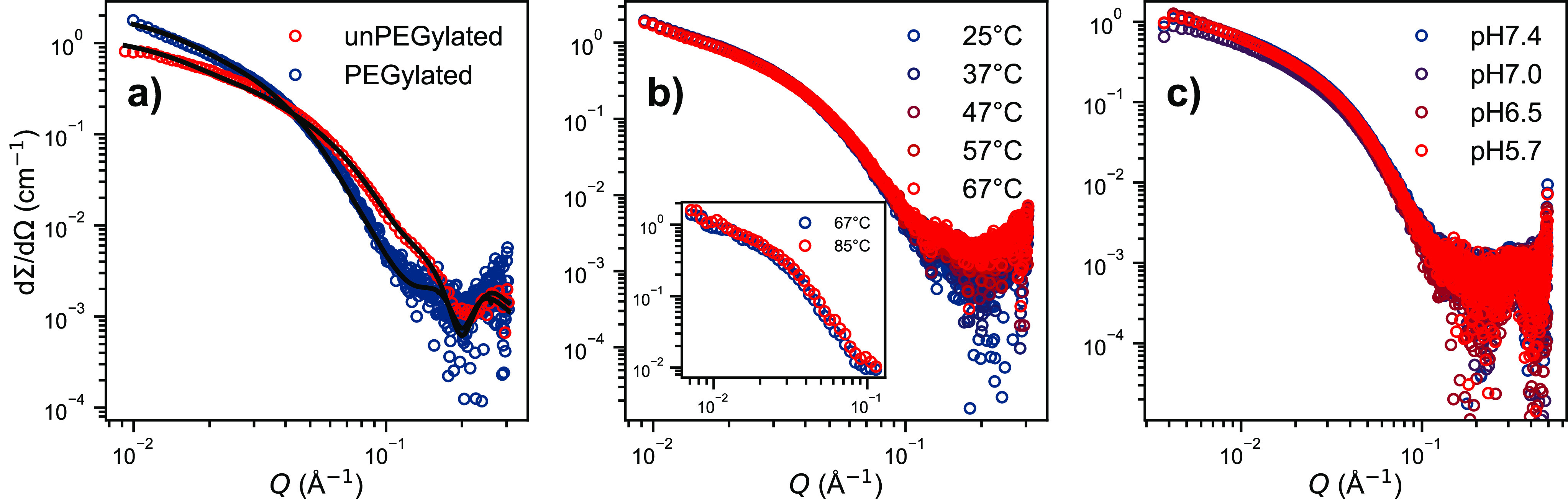
a) Scattering curves of K_3_W(QL)_6_K_2_ and PEG-K_3_W(QL)_6_K_2_ at 25 °C. Black lines represent a simultaneous fit of both data sets. b) Temperature stability of PEG-K_3_W(QL)_6_K_2_ fibers: SAXS curves at 25–67 °C and (inset) SANS curves at 67 and 85 °C. c) SAXS curves of PEG-K_3_W(QL)_6_K_2_ in BisTris buffer solution at different pH.

**Figure 5:**
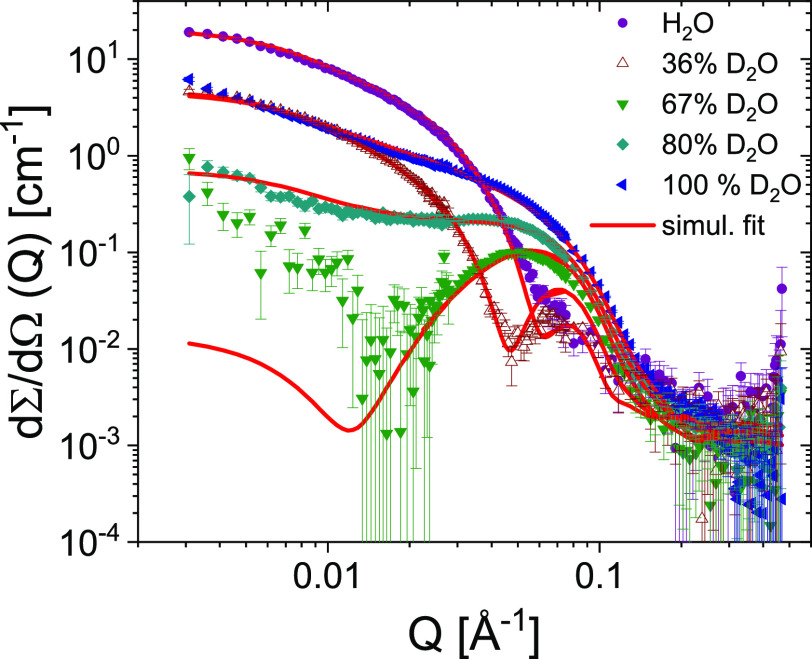
Contrast variation SANS analysis of the PEG-K_3_W(QL)_6_K_2_ nanofibers showing the scattering curves at five different H_2_O/D_2_O contrasts where various parts of the structure is highlighted. The solid lines correspond to a simultaneous fit of all scattering curves using the model described in the text.

In a previous work,^75^ Xu et al. showed that the PEGylated form of K_3_W(QL)_6_K_2_, PEG-K_3_W(QL)_6_K_2_, exhibits increased hemocompatibility, i.e., the measure of the viability of red blood cells upon treatment with different peptide assemblies. It was also found that while the internal packing was slightly disrupted by the additional entropic repulsion introduced by the PEG chains, the overall fiber structure was retained. The same observation was previously made for other PEG–peptide conjugates.^73,74^ Here we show that PEGylation does not affect the fiber stability negatively, either. Neither does it affect the structure determined via the SAXS/SANS experiments. Figure 4a shows SAXS data of K_3_W(QL)_6_K_2_ and PEG-K_3_W(QL)_6_K_2_. Both experimental curves are reproduced perfectly by the core–shell–shell model introduced in the Experimental Section, using the exact same parameters as in Table 1 for the peptide, with an additional 30 Å layer of hydrated PEG in the case of PEG-K_3_W(QL)_6_K_2_. Apparently, PEG loosely wraps around the peptide fibers, concurring with other works where PEG was found to wrap around proteins.^85^ In order to further investigate the nanostructure formed by PEG-K_3_W(QL)_6_K_2_, we performed contrast-variation SANS measurements and performed a simultaneous fit of all five data sets corresponding to each contrast. The data, depicted in Figure 5, show an excellent agreement with the scattering model and confirms rather clearly the nanostructure and specifically that PEG seems to be rather uniformly distributed at about a 30 Å layer around the fibers and not tightly wrapped to the residues as also suggested by Pai et al.^86^ for PEGylated lysozyme and human growth hormone proteins.

Even though PEGylation was previously found to slightly disrupt the internal packing of the beta-sheets,^75^ it apparently does not adversely affect the overall fiber stability as can be seen in Figure 4b and c. The SAXS pattern of PEG-K_3_W(QL)_6_K_2_ does not change between 25 and 67 °C, and the inset shows SANS data even up to 85 °C, where still no change is visible. The PEG-K_3_W(QL)_6_K_2_ peptide was also dissolved in BisTris buffers of different pH, where no change is visible down to pH 5.7, either. This demonstrates the considerable physical stability of the fibers formed by PEG-K_3_W(QL)_6_K_2_. As described in the previous section, ‘Peptide K_3_W(QL)_6_K_2_’, the attractive forces promoting self-assembly strongly dominate over the repulsive forces. They are even strong enough to counter the additional entropic repulsion introduced by PEG chains. However, in the case of the presence of destabilizing forces, like electrostatic repulsion, as shown in the SI, in Figure S7b,d,f, sequences with additional lysine residues PEG-K_4_W(QL)_6_K_2_, PEG-K_5_W(QL)_6_K_2_, and the isomer PEG-W(QL)_3_K_5_(QL)_3_ presented lower stability than their nonmodified analogues.^87^ This shows that a balance between repulsive and stabilizing attractive interactions is needed and that K_3_W(QL)_6_K_2_ exhibits an optimal composition that leads to a stable structural integrity.

### 2.5 Dynamic Stability: Molecular Exchange

Lastly, we studied the molecular exchange of PEGylated peptide molecules between peptide fibers. To this end, we made use of the kinetic-zero-average-contrast scheme,^88^ an advanced SANS experiment that has been previously applied to study the molecular exchange of polymer micelles.^35^ This method allows for the determination of the exchange processes: fusion/fission or single-molecule (unimer) diffusion taking place between the assembled entities. Here we employed hPEG-K_3_W(QL)_6_K_2_ and dPEG-K_3_W(QL)_6_K_2_, K_3_W(QL)_6_K_2_ conjugated with proteated and deuterated PEG. The two species were separately dissolved in 50 mM Tris buffer (56% D_2_O, 44% H_2_O), which has a scattering length density exactly in between those of h- and dPEG-K_3_W(QL)_6_K_2_. This way, the two peptide species have the same overall contrast but with opposite sign. The two solutions were thoroughly mixed, and the blended solution was measured with SANS over time. As the two fiber populations exchange molecules with deuterated and proteated PEG chains, the PEG shell becomes isotopically mixed so that its contrast against the solvent decreases. Thus, the exchange of molecules is manifested by a decay in the scattering intensity over time.

**Figure 6:**
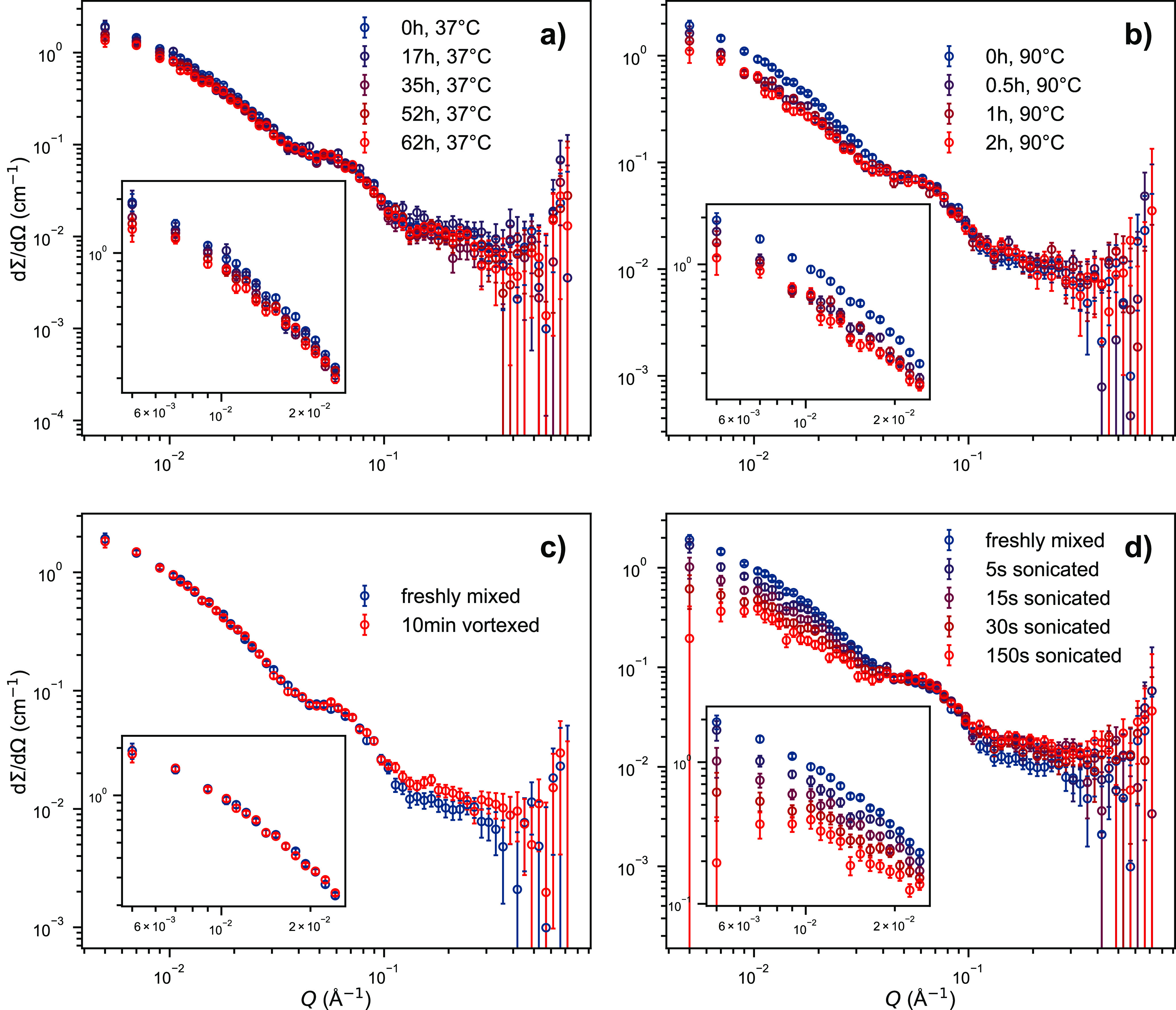
Neutron scattering data of blends of deuterated and protonated PEG-K_3_W(QL)_6_K_2_ in a zero-average-contrast Tris buffer (pH 7.4 at 25 °C, 56% D_2_O). Molecular exchange is visible as a decrease in scattering intensity. The insets are magnifications of the low-*Q* region. a) Thermally activated exchange at 37 °C. b) Thermally activated exchange after storing the blended sample in an oven at 90 °C for the indicated duration. c) Mechanically activated exchange using a benchtop vortex mixer. d) Mechanically activated exchange using a tip-sonicator.

Figure 6 shows time-resolved neutron scattering curves of such blends, with the insets showing magnifications of the low-*Q* region. We first investigated the thermally activated exchange of molecules. As can be seen in Figure 6a), after 2.5 days at 37 °C there is barely any reduction in scattering intensity. Also at elevated temperatures up to 67 °C, there was virtually no change in the scattering signal. Therefore, we finally increased the temperature to 90 °C, after testing that the fibers do not dissolve at such high temperatures (see Figure 4b). Since it was technically not possible to heat the sample holder to this temperature, the cuvette with the freshly blended peptide solution was sealed with tape and stored in an oven at 90 °C for the desired duration. Then the cuvette was measured at 37 °C before it was put in the oven again. As there is practically no exchange at 37 °C, the transport between oven and experimental hutch as well as the measurement duration itself are negligible. Interestingly, the scattering intensity decreased significantly after only 30 min at 90 °C but then stayed roughly constant, even after another 90 min in the oven. We therefore hypothesize that the thermally activated molecular exchange happens only via the molecules at the very ends of the fibers. This is thermodynamically more favorable than removing molecules from the middle of the beta-sheets, which would require breaking twice as many hydrogen bonds. Also breaking the whole fiber in two (molecular exchange via fiber break-up and reformation) involves breaking both stacked beta-sheets simultaneously, which requires breaking twice as many hydrogen bonds as well. The consequence of exchange happening only at the terminal positions of the beta sheets is that, while the fiber ends rather quickly become isotopically mixed, the inner parts of the fibers will rarely see molecular exchange as it takes more time for the ’exchange front’ to advance along the fiber contour. But even the proposed single-molecule exchange mechanism at the fiber ends is surprisingly slow at physiological temperature.

To test if any molecular exchange could be provoked, we subjected the peptide fibers to mechanical agitation, inspired by a similar study on polymer micelles.^89^ A total of 10 min vortex mixing, as described in the Experimental Section, did not induce any exchange: compare Figure 6c. We therefore turned to a more disruptive method, tip-sonication. This technique is often used when noncovalently bound but physically stable structures need to be broken up, for instance lysing cell membranes in protein expression protocols. Indeed, mechanical agitation using a tip-sonicator did induce molecular exchange (see Figure 6d). Obviously, the exchange does not depend linearly on the sonication duration. This might be explained by the fact that the sample exhibited an increasing amount of foam during sonication, which likely reduced the sonication efficiency over time. Nevertheless, tip-sonication seems to be an effective way to induce molecular exchange, presumably by breaking apart the peptide fibers. These findings prove that neutron scattering in combination with (partial) deuteration schemes is in principle applicable to investigate the molecular exchange kinetics of peptide assemblies. It should be noted that tip-sonication is a rather rough treatment, but all samples were checked with SAXS after the kinetic experiments, showing no altered scattering pattern compared to untreated samples.

We can rationalize the strong binding of peptide molecules to the assembled fibers by a rough empirical estimation. The first major driving force of assembly is the hydrophobic effect, where each peptide molecule contains 24 aliphatic carbon atoms from its leucine residues. The activation energy of molecular exchange in a related system, n-alkyl-PEG amphiphiles forming spherical micelles, was determined by Zinn et al. from TR-SANS,^90^ yielding approximately 90 kJ/mol for 24 aliphatic carbon atoms. Alternatively, using Tanford’s^91^ values of 9 kJ/mol per CH_3_ and 3 kJ/mol per CH_2_ group, results in an even higher value for the hydrophobic contribution, that is 144 kJ/mol for the six leucine residues per molecule. The second major binding factor is hydrogen bonds between the peptide backbones. The MD simulations show that the central KW(QL)_6_ block forms stable hydrogen bonds, resulting in 14 bonds on each side of a peptide molecule. With each contributing about 8 kJ/mol binding energy,^92^ this results in 112 kJ/mol for removing a molecule from the very end of a fiber. Together with the hydrophobic interaction, these estimations yield a very high activation energy of molecular exchange of at least 200 kJ/mol. This rough estimate thus illustrates the strong binding of K_3_W(QL)_6_K_2_ molecules in the fiber assemblies. The calculation, of course, does not consider the electrostatic repulsion between the lysine residues, but neither does it consider the hydrophobic contribution of tryptophan nor the attractive interactions (hydrogen bonds and electrostatics) between glutamine residues. This empirical estimate agrees with the per-residue energetic contribution of the peptide unimer ligand obtained from the simulations, where the central KW(QL)_6_ block has an energetic contribution of ∼203 kJ/mol, while the lysine residues are unfavorable to binding (≈ −57 kJ/mol). The rough overall estimate is also in fair agreement with the estimate of total binding free energy of 319.4 ( ±27.7) kJ/mol calculated with the MM-GBSA approach. However, that value is somewhat uncertain, as it does not include entropic contributions to binding energy. Nevertheless, we can conclude that the great physical integrity of the peptide fibers is due to the many attractive interactions between peptide strands overcoming the repulsive interactions of the lysine residues.

In comparison, da Silva et al.^39^ have observed molecular exchange of unimers and small clusters of molecules between fibers of n-alkyl-peptide conjugates on the time scale of hours. This difference can be attributed to the different molecular structure; in the present case, the hydrophobic interaction and hydrogen bonds act in concert to stabilize the beta-sheet structure, whereas the alkylated peptides would behave more like an amphiphile with added stability from the hydrogen bonds in the beta-sheet. The molecules investigated in the study by da Silva et al. also contain only 13 aliphatic carbons (24 in K_3_W(QL)_6_K_2_) and form a maximum of 9 hydrogen bonds (14 in K_3_W(QL)_6_K_2_), so they are less strongly bound.

The present study provides insight into the factors determining the stability of self-assembled peptide nanostructures. It is important for the general understanding of self-assembled systems and peptide assemblies, in particular, that can be used as biomaterials or delivery vehicles in both nano- and bio-technological applications.

## 3 Conclusions

In the present work, we used small-angle scattering techniques in combination with molecular dynamics simulations to show that fibers formed by K_3_W(QL)_6_K_2_ multidomain peptides are extraordinarily stable. Both PEGylated and non-PEGylated versions of the K_3_W(QL)_6_K_2_ peptide exhibited no detectable morphology change under elevated temperature (up to 85 °C), acidic conditions (down to pH 5.0), and presence of a denaturing agent (4 M urea). To analyze the data, we presented a geometric core–shell model that yields a better description of the peptide scattering data than previously used models. The data also indicate that the PEG forms a solvent-swollen shell around the peptide fiber. In addition, we investigated the molecular exchange of PEGylated peptide fibers using a contrast-variation neutron scattering scheme. We found the thermally activated exchange of molecules between fibers to be unexpectedly slow and explain this finding by the exceptional stability of the stacked beta sheets, so that practically only molecules from the ends of the fibers exchange. This finding is in line with a very high binding energy of the peptide molecules as determined from molecular dynamics simulations. We did, however, trigger molecular exchange via mechanical agitation. While vortex mixing had no measurable effect, tip-sonication caused the self-assembled fibers to break up and reform. We therefore showed that the kinetic-zero-average-contrast scheme is a viable technique to monitor the molecular exchange of self-assembling peptides, an important factor in the assessment of structural integrity. Additionally, analysis of the similar peptides K_4_W(QL)_6_K_2_, K_5_W(QL)_6_K_2_, and W(QL)_3_K_5_(QL)_3_ revealed that the presence of a β-sheet stabilizing motif (QL)_*y*_ is crucial for the overall stability of the nanofibers, and the observed stability of the supramolecular structure is a result of a balance between repulsive electrostatic and attractive hydrogen bonds and hydrophobic interactions.

Our study provides important insight into interactions governing peptide self-assembly. Importantly, we find that relatively short peptide sequences can form exceptionally stable superstructures. The stabilizing factors are mainly hydrophobic interactions that act in concert with hydrogen bonds. This combination bears some resemblance to peptides and proteins related to so-called plaque in Alzheimer’s disease as well as structures found in proteins associated with thermophile bacteria. For biomedical applications, self-assembling materials offer the advantages of facile preparation of well-defined structures, biocompatibility, and biofunctionality. While self-assembling approaches generally suffer from intrinsic instability, molecular exchange, and rearrangement, our findings are important for the design of self-assembling peptides where physical stability is key. These nanostructures are important in, e.g., application of delivery systems and as antibiotics where the assemblies are more resistant toward enzymatic degradation and nonspecific protein clearance providing longer blood circulation and *in situ* stability in, e.g., wound healing formulations and toward bacterial infections.^93^

## 4 Experimental Section

### 4.1 Synthesis

**Scheme 1:**
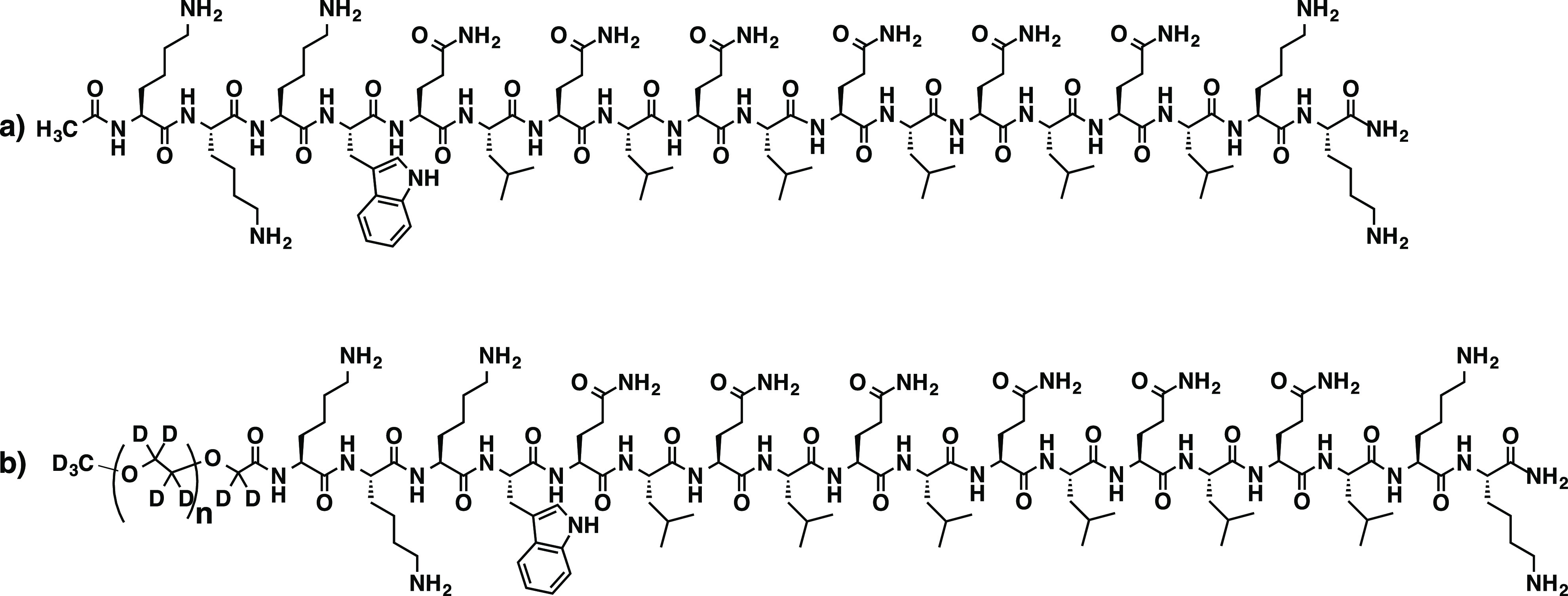
Molecular Structure of a) K_3_W(QL)_6_K_2_ and b) dPEG-K_3_W(QL)_6_K_2_

All peptides were synthesized on a prelude peptide synthesizer using standard Fmoc solid-phase peptide synthesis. 20% (V/V) piperidine in DMF was used to deprotect Fmoc groups. HCTU and DIPEA were used as amino acid coupling reagents in a molar ratio of 1:1:2.5 (amino acid: HCTU: DIPEA). Fmoc protected amino acids were added in 4 equiv to the resin. The N-terminus was acetylated in the presence of 50 equiv of acetic anhydride and 6 equiv of DIPEA in DMF. For PEGylated peptide, 4 equiv of PEG-COOH was added to the resin and allowed to react overnight. Regular proteated PEG-OH (1.9 kg/mol) was bought from Sigma-Aldrich and deuterated PEG-OH (2.1 kg/mol) was synthesized via ring-opening living anionic polymerization of deuterated ethylene oxide as described by König et al.^94^ The terminal −CH_2_(CD_2_)–OH groups of the PEG polymers were converted into carboxy groups by TEMPO-mediated oxidation according to a procedure described by Araki et al.^95^ Kaiser test was used to confirm the completion of the peptide-polymer coupling reaction. The peptides were cleaved from the resin in a mixture of TFA/Tris/water (95:2.5:2.5 by volume) for 3 h. TFA solution was collected, and the resin was rinsed twice with neat TFA. After evaporation of the combined TFA solutions, the residual peptide solution was triturated with chilled diethyl ether. The resulting precipitate was centrifuged and washed three times with a chilled diethyl ether. The crude peptide was dried under a vacuum overnight for dialysis. The dialyzed peptide was subsequently lyophilized to get purified peptide powder and the mass of each peptide was confirmed by MALDI-TOF mass spectrometry using α-cyano-4-hydroxycinnamic acid as the matrix (see Supporting Information). Scheme 1 shows the molecular structure of K_3_W(QL)_6_K_2_ and its dPEG-conjugated (deuterium-labeled) counterpart dPEG-K_3_W(QL)_6_K_2_.

### 4.2 Sample Preparation

Dry peptide lyophilizate was weighed and dissolved in the desired amount of buffer solution. If necessary, the samples were shaken gently at room temperature to facilitate dissolution. If not noted otherwise, samples presented in this manuscript were dissolved in a 50 mM Tris (pH 7.4) buffered solution with a target concentration of 10 mg/mL, corresponding to a volume fraction of about ϕ ≈ 0.8%. For pH-dependent studies, 25 mM BisTris (pH 7.4, 7.0, 6.5, and 5.7) or 20 mM MES (pH 5.0) were used instead. For evaluation of the influence of urea, standard solutions of peptides were mixed in proportion 1:1 v:v with 8 M urea solution containing 50 mM Tris, resulting in 5 mg/mL peptide solution in 4 M urea and 50 mM Tris.

### 4.3 SAXS

Small-angle X-ray scattering (SAXS) experiments were performed at two instruments, the synchrotron beamline BM29^96−98^ at the European Synchrotron Radiation Facility in Grenoble, France, and an in-house Bruker NanoStar located at the University of Oslo, Norway. At BM29, a photon wavelength of λ = 1 Å was used at a sample-to-detector distance of 2.9 m, resulting in accessible wave vectors *Q* = 0.004–0.5 Å^–1^, where  and θ is the scattering angle. To exclude beam damage, exposure was split into ten frames, which were averaged if no systematic deviation was detected. Buffer measurements were taken both before and after the sample and then were averaged as well. The experiments in Oslo were performed using Cu *K*_α_ radiation (λ = 1.54 Å) and an available *Q* range of 0.01–0.3 Å^–1^. Because of the low X-ray flux, no particular attention has to be paid to possible radiation damage here. Data reduction on both instruments was performed according to the instrument standard protocols.

### 4.4 SANS

Small-angle neutron scattering (SANS) experiments were performed on two different instruments: KWS-2^99^ at the Heinz Maier-Leibnitz Zentrum in Garching, Germany, and Sans2d^100−102^ at the ISIS Pulsed Neutron and Muon Source in Didcot, United Kingdom. At KWS-2, neutrons with 5 Å wavelength and sample-to-detector distances of 4 m and 8 m were used, resulting in a combined *Q* range of 0.01–0.5 Å^–1^. At the time-of-flight instrument Sans2d, neutrons with 1.75 ≤ λ ≤ 16.5 Å and a single sample-to-detector distance of 4 m covered *Q* = 0.005–0.7 Å^–1^. The data from both experiments were reduced according to the instrument standard protocols.

### 4.5 Mechanical Agitation

To induce peptide fiber break-up and reformation, samples were subjected to mechanical agitation. A commercially available benchtop vortex mixer (FisherScientific TopMix FB15024) was used at maximum intensity. Samples were agitated for a maximum of 1 min at a time, followed by a 1 min minimum rest to avoid sample heating. Tip-sonication was performed using a FisherScientific FB50 device at a 20% intensity. Sonication was split into pulses of 5 s followed by a 10 s rest to avoid sample heating.

### 4.6 Scattering Models

**Figure 7:**
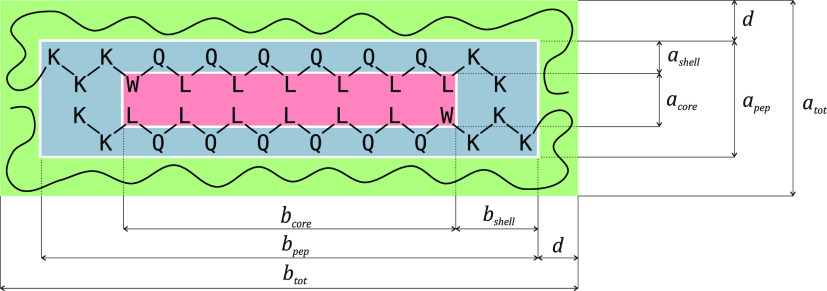
Sketch to illustrate the cross-section of the peptide scattering model. The core (red) is formed by the hydrophobic residues, which are shielded from the solvent by the peptide backbone and the hydrophilic residues (blue). In the case of the PEGylated peptide, PEG presumably forms a second, solvent-swollen shell around the peptide (green).

To interpret the scattering data, a geometrical model of the peptide has been developed. It is based on a simpler model that has previously been used.^60,75^ The peptide self-assembles into long fibers, consisting of two stacked antiparallel beta-sheets. Between the two beta-sheets, the leucine residues form a hydrophobic core, which is probably also occupied by tryptophan residues. The peptide backbone and hydrophilic residues shield the core from the solvent. This morphology motivates a core–shell structure to model the peptide. In addition, in the case of the PEGylated peptide, the PEG chains can as a first approximation be assumed to form a third, homogeneous solvent-swollen shell around the peptide. Because the fiber length, defined as *c*, is much greater than the other two dimensions, (a,b), the longitudinal dimension can be decoupled from the peptide cross-section. Figure 7 shows a sketch of the peptide cross-section according to the model.

The core, formed by leucine and tryptophan residues, has cross-sectional dimensions *a*_core_ and *b*_core_. The shell encompasses the peptide backbone as well as the hydrophilic residues and has the thicknesses *a*_shell_ and *b*_shell_ so that the peptide cross-section has the dimensions *a*_pep_ = *a*_core_ + 2*a*_shell_ and *b*_pep_ = *b*_core_ + 2*b*_shell_. Finally, in the case of the PEGylated peptide, we assume a second shell of thickness *d* which includes the solvent-swollen PEG. The scattering amplitudes of the different components are calculated via the scattering amplitude of a simple rectangle:^103^
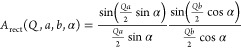
1Here, *Q* is the scattering vector and the expression needs to be integrated over α for a rotational average. From this, the three cross-sectional scattering amplitudes are calculated as
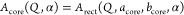
2a
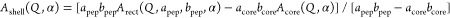
2b
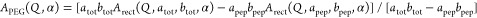
2cwhere *a*_tot_ = *a*_pep_ + 2*d* and *b*_tot_ = *b*_pep_ + 2*d* are the dimensions of the total structure. In the case of the non-PEGylated peptide, *A*_PEG_(*Q*, α) is simply set to zero. Weighted by the respective contrasts *Δρ* and volumes, the overall cross-sectional amplitude is
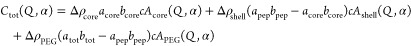
3where *c* ≫ *a*_tot_, *b*_tot_ is the fiber length. The longitudinal scattering is given by that of an infinitely thin rod:^104^
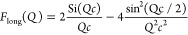
4a
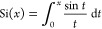
4bTherefore, the total scattering from the rectangular peptide filament is
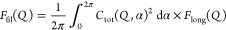
5In addition, the model accounts for density fluctuations within the PEG shell, the so-called ‘blob scattering’.^105^ This is done via a simple Beaucage form factor^106^ with radius of gyration *R*_*g*_:
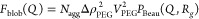
6where *N*_agg_ is the number of molecules per fiber, calculated from the fiber dimensions and the peptide molecular volume, and *V*_PEG_ is the PEG molecular volume. Finally, the total scattering intensity is calculated using the volume fraction ϕ as
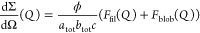
7Consideration of polydispersity along the longitudinal axis did not improve fit results; an expected result given that the relevant length scale is on the edge of the experimental resolution. Therefore, polydispersity was ignored in order to minimize the number of fit parameters.

We note that we initially tried to refine existing scattering models^21,107^ to our experimental data. The resulting fits were poor, though, particularly in terms of refining against SAXS and SANS data simultaneously; therefore, we resorted to developing our own model tailored to the sample system.

### 4.7 Molecular Dynamics Simulations

A 200 strand non-PEGylated K_3_W(QL)_6_K_2_ peptide fiber (100 strands per sheet) was assembled in VMD^108^ with a geometry designed to maximize interstrand hydrogen bonding. The assembly was placed in neutralizing ions and TIP3P^109^ water with at least 15 Å of additional water in all directions to prevent interactions across the periodic boundary. Simulations were minimized for 50000 steps using the conjugate gradient method and then equilibrated for 15 ns at 300 K in the NPT ensemble with a 1 fs time step. All simulations were run in NAMD^110^ with CHARMM36^111^ force field parameters, and the long-range electrostatics were calculated with the Particle Mesh Ewald method^112^ with a cutoff of 12 Å. Snapshots taken at the end of the equilibration were used to predict the theoretical SAXS/SANS scattering curve with CRYSOL^79^ and CRYSON^80^ from the ATSAS software suite,^113^ respectively, though it was found that the predicted SAXS/SANS curves changed very little over the last 5 ns of the equilibration.

Additionally, an estimate of binding energy was made using the molecular mechanics generalized Born surface area (MM-GBSA) single trajectory method over the last 2.5 ns of the simulation. MMPBSA.py was used to perform the calculations.^114^ The receptor was defined as a 99 strand K_3_W(QL)_6_K_2_ sheet and the adjacent 100 strand sheet, while the ligand was defined as the remaining peptide unimer. Together, these selections define the peptide complex, which is composed of 200 peptide nanofiber. A salt concentration of 0.01 M was selected with an interior and exterior dielectric constant of 1 and 80, respectively. As the calculation of the entropic contribution to binding free energy is challenging^115^ and can be prone to large error, it was ignored as has been done previously.^116,117^ Finally, the molecular volume of the model was calculated using ProteinVolume 1.3.^118^

### 4.8 Circular Dichroism

Circular dichroism (CD) spectroscopy of (100 μM peptide solutions in 20 mM Tris buffer, pH 7.4 and 6.5, 20 mM MES buffer, pH 5.7 and 5.0) was performed on a Jasco-710 spectrometer. The data shown in Figure S6 are averages of ten scans from 250 to 195 nm, obtained at room temperature with a scan rate of 100 nm/min, a bandwidth at 0.1 nm, and a response time of 2 s. The raw data were converted to molar residual ellipticity via
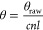
8where *c* = 0.1 mM is the peptide concentration, *n* the number of residues per peptide molecule, *n* ∈ {18, 19, 20}, and *l* = 1 mm the optical path length of the sample cuvette. Analysis of the CD spectra was performed using the Beta Structure Selection program (BeStSel)^119,120^

## Supporting Information Available

HPLC, MALDI-TOF results, pH-dependent circular dichroism spectra, additional scattering curves for K_4_W(QL)_6_K_2_, K_5_W(QL)_6_K_2_, W(QL)_3_K_5_(QL)_3_, PEG-K_4_W(QL)_6_K_2_, PEG-K_5_W(QL)_6_K_2_, PEG-W(QL)_3_K_5_(QL)_3_, comparison between scattering curves calculated from MD structure and experimental data (PDF)
