# Supplementary material for: Stability of Nanopeptides: Structure and Molecular Exchange of Self-assembled Peptide Fibers
Source: ACS Nano. 2023 Jun 26;17(13):12394–408. doi: 10.1021/acsnano.3c01811 (PMC10339792; doi:10.1021/acsnano.3c01811)
Supplement: Supplementary file 1 — nn3c01811_si_001.pdf [file nn3c01811_si_001.pdf]

Supplementary Information
for

On the Stability of Nanopeptides: Structure and Molecular Exchange of Self-assembled Peptide Fibers

Nico König,[1,2] Szymon Mikolaj Szostak,[1] Josefine Eilsø Nielsen,[1,3] Martha Dunbar,[4] Su Yang,[5] Weike Chen,[5] Ari Benjamin,[4] Aurel Radulescu,[6] Najet Mahmoudi,[7] Lutz Willner,[2] Sinan Keten,[4,8] He Dong,[5] and Reidar Lund[1,9,*]

[1]*Department of Chemistry, University of Oslo, P.O. Box 1033 Blindern, 0315 Oslo, Norway*
[2]*Jülich Centre for Neutron Science (JCNS) and Institut für Biologische Informationsprozesse (IBI), Forschungszentrum Jülich GmbH, 52425 Jülich, Germany*
[3]*Current address: Department of Bioengineering, Stanford University, Stanford, California 94305, United States*
[4]*Department of Mechanical Engineering, Northwestern University, Evanston, Illinois 60208, United States*
[5]*Department of Chemistry & Biochemistry, The University of Texas at Arlington, Arlington, Texas 76019, United States*
[6]*Jülich Centre for Neutron Science (JCNS) at Heinz Maier-Leibnitz Zentrum (MLZ), Forschungszentrum Jülich GmbH, 85747 Garching, Germany*
[7]*ISIS-STFC, Rutherford Appleton Laboratory, Chilton, Oxon OX11 0QX, United Kingdom*
[8]*Department of Civil and Environmental Engineering, Northwestern University, Evanston, Illinois 60208, United States*
[9]*Hylleraas Centre for Quantum Molecular Sciences, University of Oslo, Norway*

HPLC AND MALDI-TOF CHARACTERIZATION

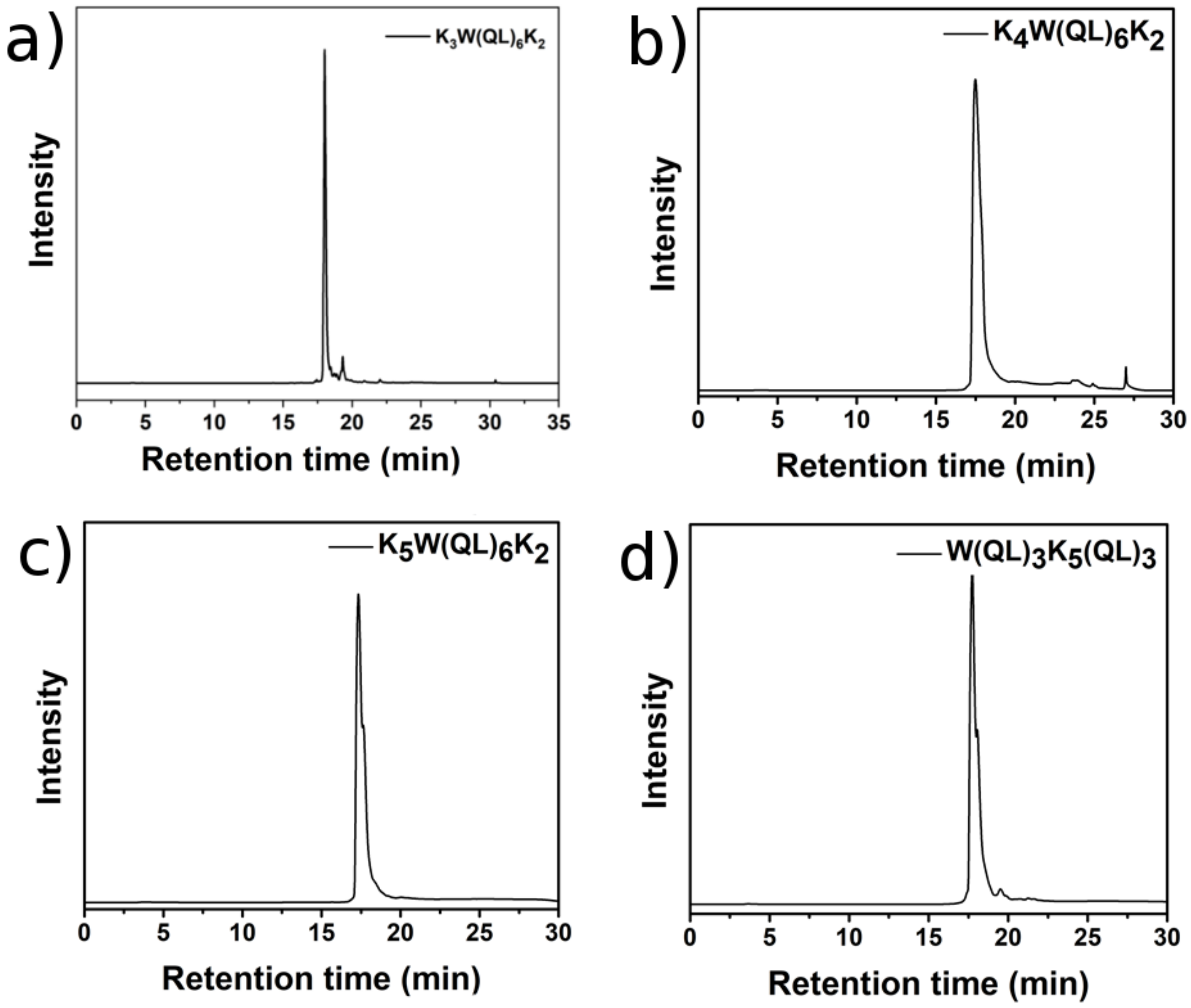

FIG. S1: a) HPLC chromatogram of $K_3W(QL)_6K_2$, b) HPLC chromatogram of $K_4W(QL)_6K_2$, c) HPLC chromatogram of $K_5W(QL)_6K_2$, d) HPLC chromatogram of $W(QL)_3K_5(QL)_3$.

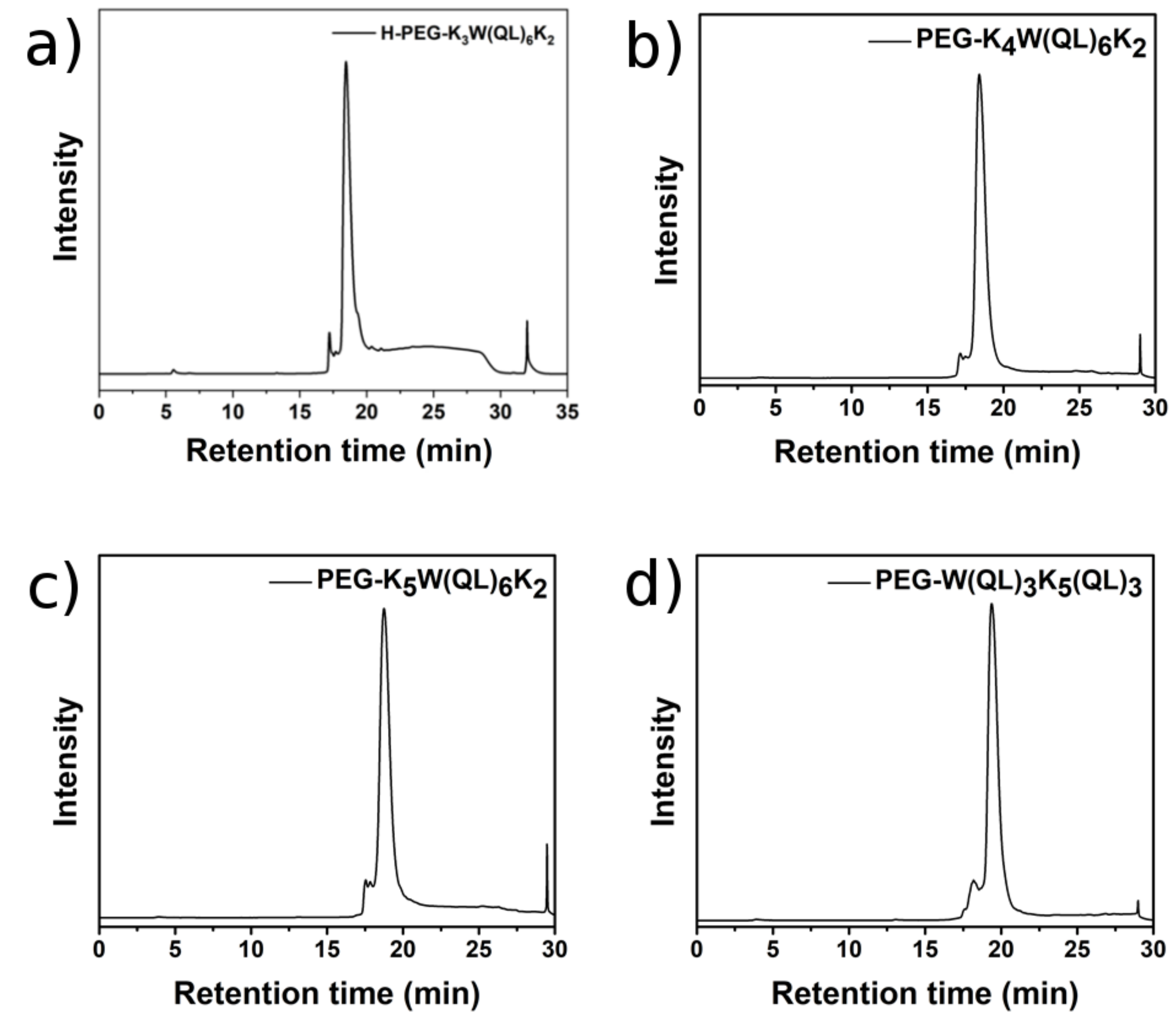

FIG. S2: a) HPLC chromatogram of PEG-$K_3W(QL)_6K_2$, b) HPLC chromatogram of PEG-$K_4W(QL)_6K_2$, c) HPLC chromatogram of PEG-$K_5W(QL)_6K_2$, d) HPLC chromatogram of PEG-$W(QL)_3K_5(QL)_3$.

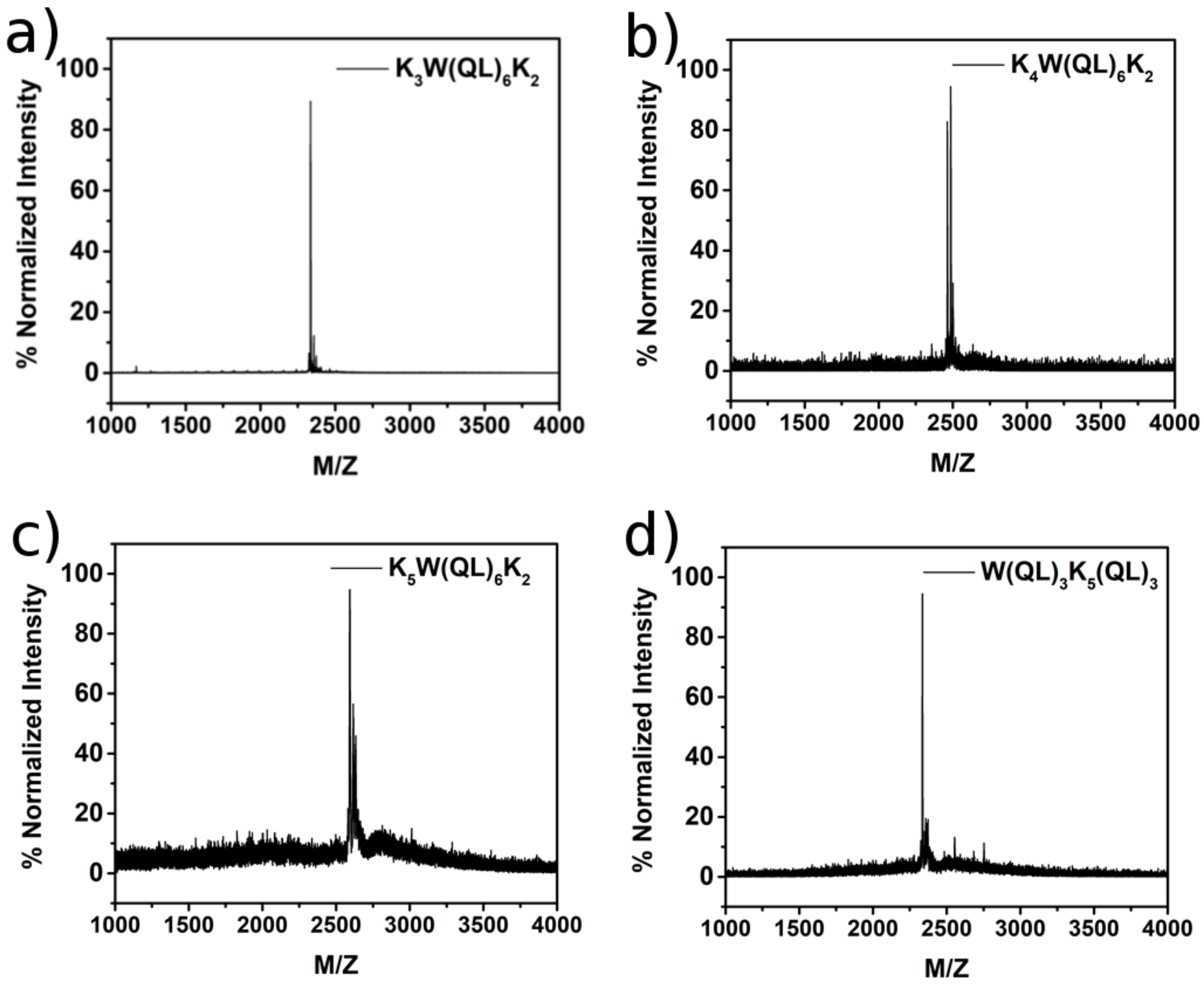

FIG. S3: a) MALDI-TOF spectrum of $K_3W(QL)_6K_2$, b) MALDI-TOF spectrum of $K_4W(QL)_6K_2$, c) MALDI-TOF spectrum of $K_5W(QL)_6K_2$, d) MALDI-TOF spectrum of $W(QL)_3K_5(QL)_3$

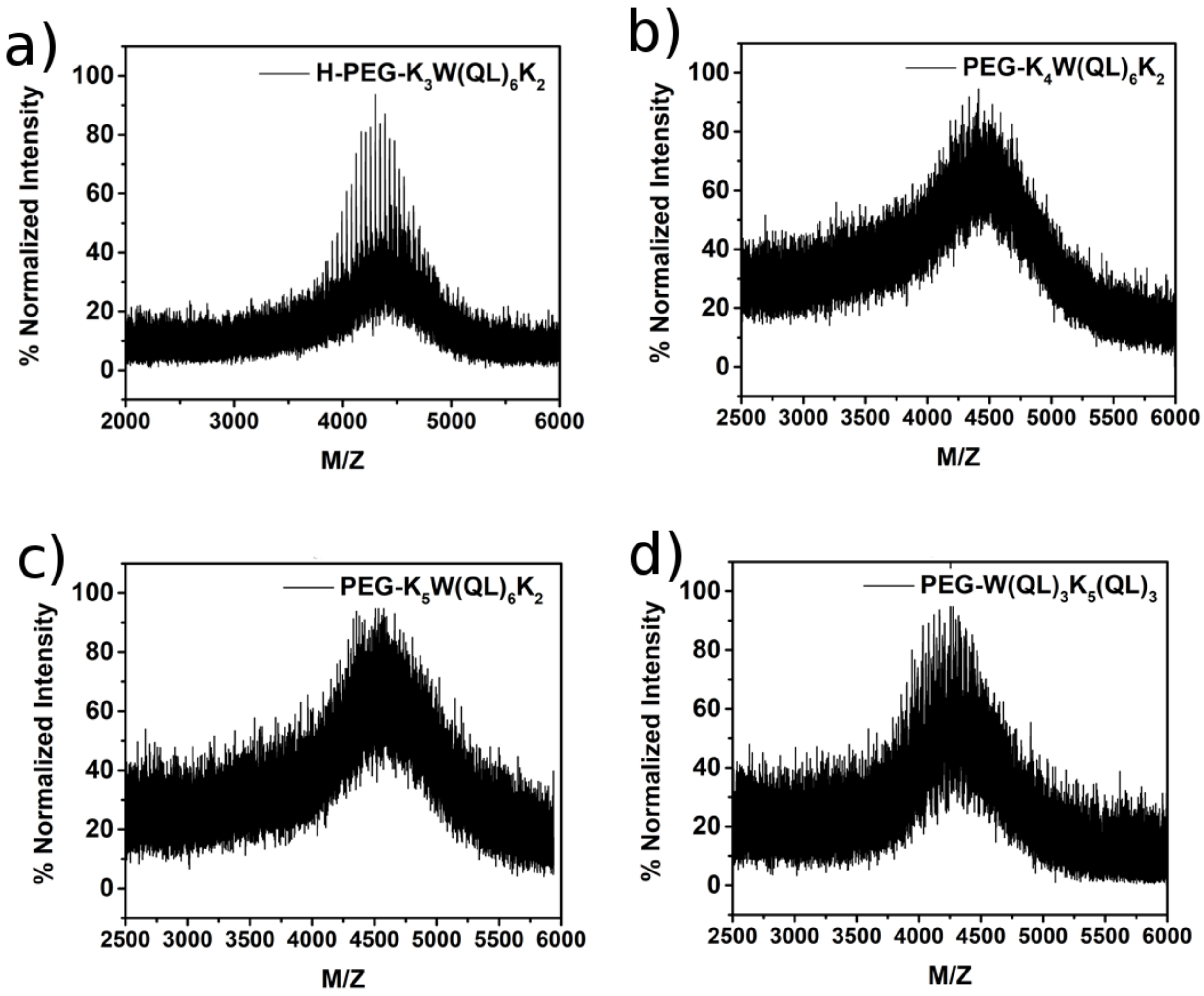

FIG. S4: a) MALDI-TOF spectrum of PEG-$K_3W(QL)_6K_2$, b) MALDI-TOF spectrum of PEG-$K_4W(QL)_6K_2$, c) MALDI-TOF spectrum of PEG-$K_5W(QL)_6K_2$, d) MALDI-TOF spectrum of PEG-$W(QL)_3K_5(QL)_3$

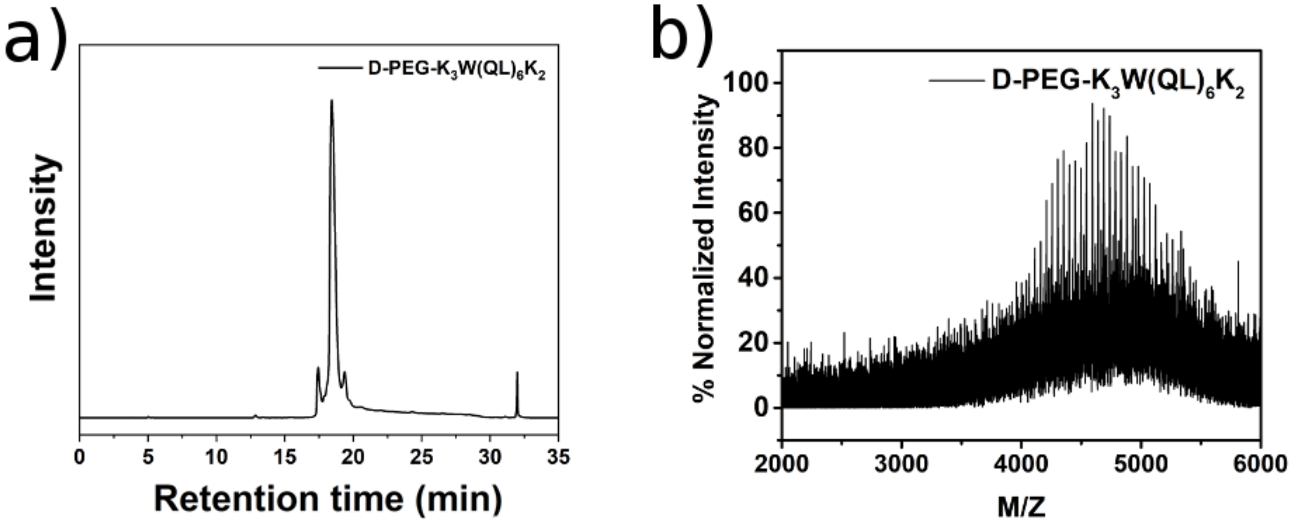

FIG. S5: a) HPLC chromatogram of dPEG-$K_3W(QL)_6K_2$, b) MALDI-TOF spectrum of dPEG-$K_3W(QL)_6K_2$.

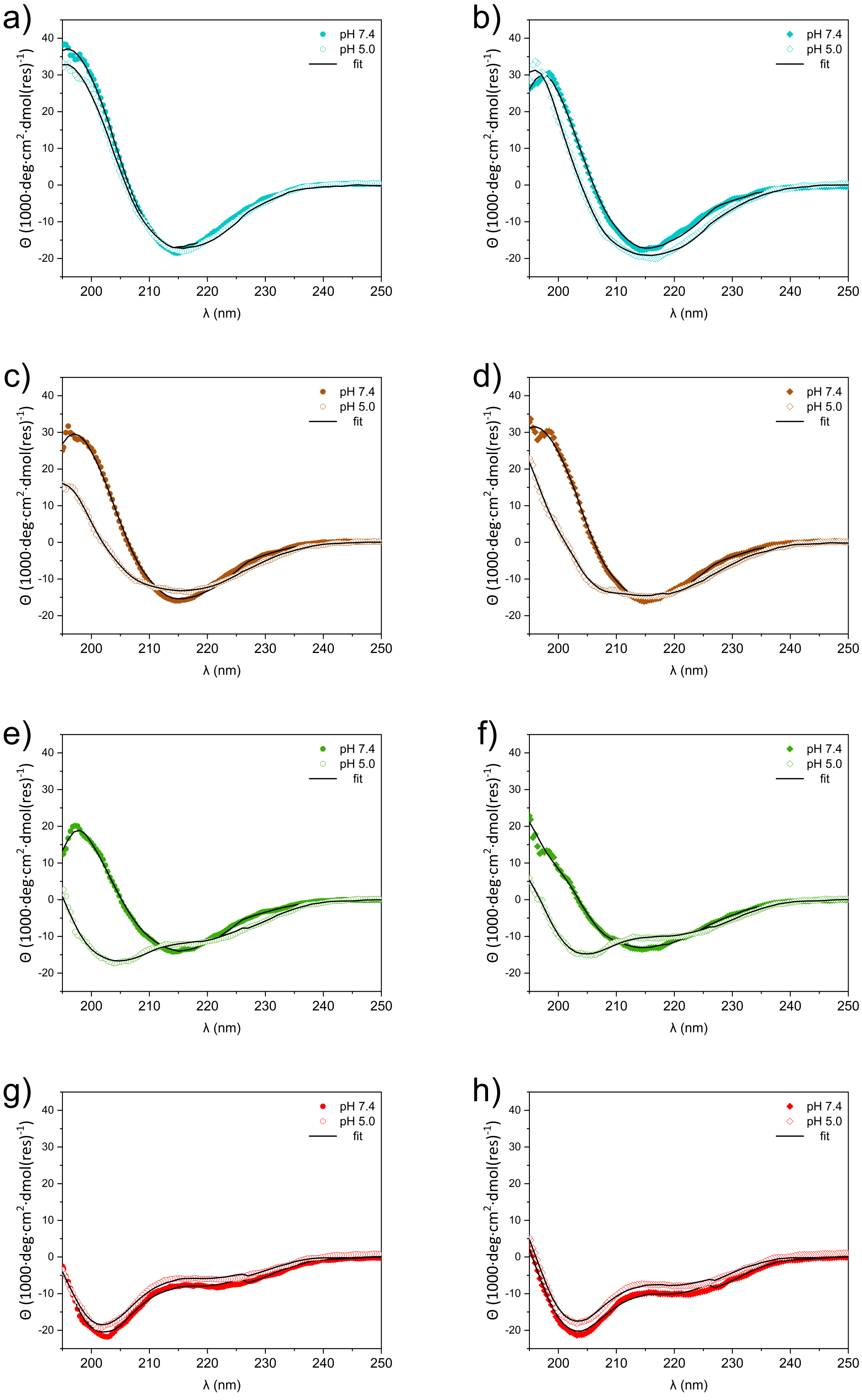

FIG. S6: CD spectra of peptide solutions at pH 7.4 and 5.0, black lines represent CD spectrum matched by BeStSel program a) $K_3W(QL)_6K_2$, b) PEG-$K_3W(QL)_6K_2$, c) $K_4W(QL)_6K_2$, d) PEG-$K_4W(QL)_6K_2$, e) $K_5W(QL)_6K_2$, f) PEG-$K_5W(QL)_6K_2$, g) $W(QL)_3K_5(QL)_3$, h) PEG-$W(QL)_3K_5(QL)_3$

TABLE S1: Summary of the estimated secondary structure content in the peptide nanostructures, determined by BeStSel program and represented in Figure S6

Peptide		Estimated secondary structure content [%]		
		α-helix	β-sheet	Other
$K_3W(QL)_6K_2$	pH = 7.4	18	69	13
	pH = 5.0	21	66	13
$K_4W(QL)_6K_2$	pH = 7.4	18	54	28
	pH = 5.0	21	29	50
$K_5W(QL)_6K_2$	pH = 7.4	19	31	50
	pH = 5.0	20	22	58
$W(QL)_3K_5(QL)_3$	pH = 7.4	12	20	68
	pH = 5.0	10	18	72
PEG-$K_3W(QL)_6K_2$	pH = 7.4	16	44	40
	pH = 5.0	31	27	42
PEG-$K_4W(QL)_6K_2$	pH = 7.4	15	67	18
	pH = 5.0	20	48	32
PEG-$K_5W(QL)_6K_2$	pH = 7.4	12	61	27
	pH = 5.0	20	15	65
PEG-$W(QL)_3K_5(QL)_3$	pH = 7.4	19	16	65
	pH = 5.0	17	16	67

TEMPERATURE STABILITY OF PEPTIDES

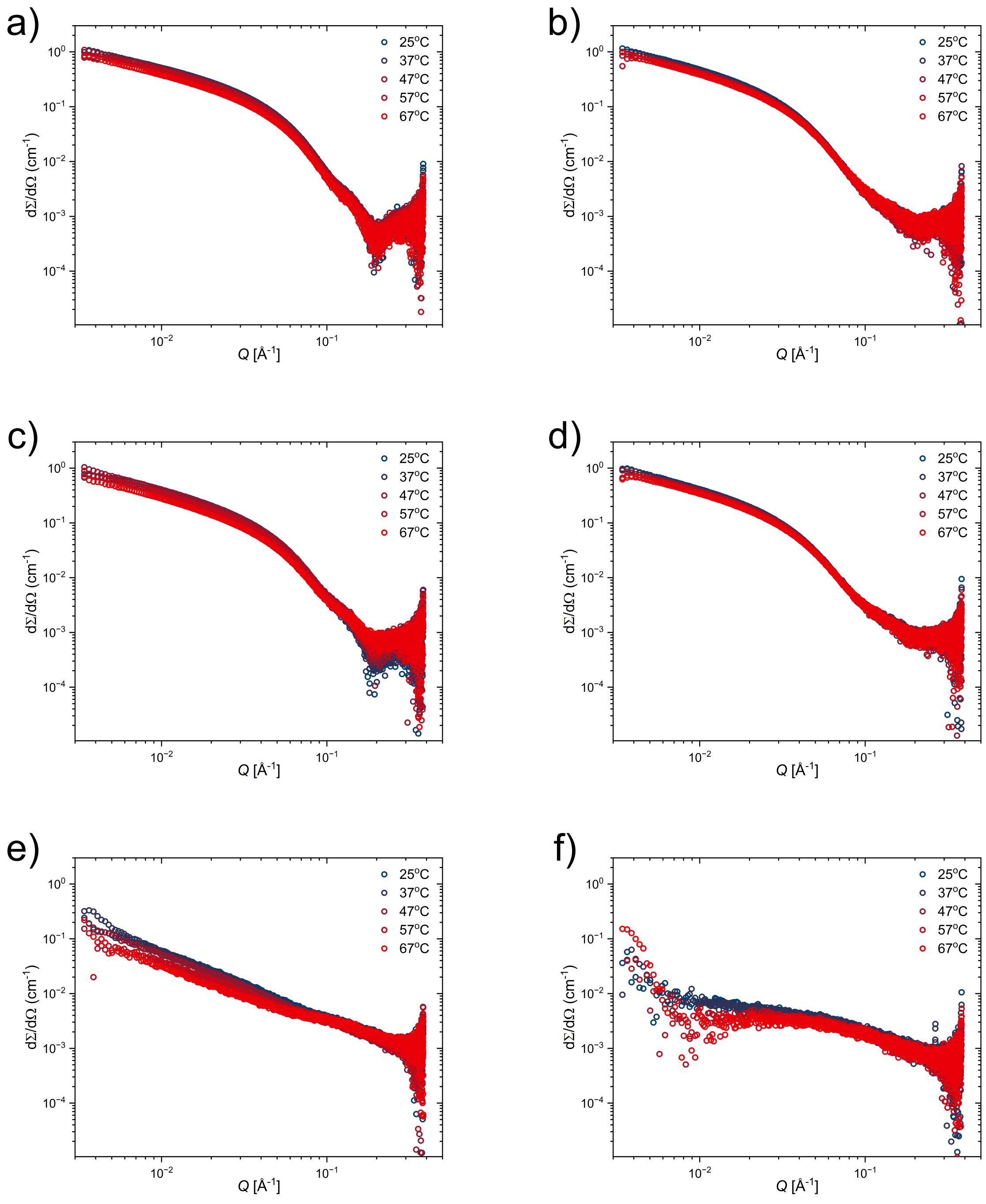

FIG. S7: The scattering data of 5 mg/ml peptide solutions in the range 25 °C - 67 °C, a) $K_4W(QL)_6K_2$, b) PEG-$K_4W(QL)_6K_2$, c) $K_5W(QL)_6K_2$, d) PEG-$K_5W(QL)_6K_2$, e) $W(QL)_3K_5(QL)_3$, f) PEG-$W(QL)_3K_5(QL)_3$

CRYSOL/CRYSON CALCULATED SCATTERING CURVES

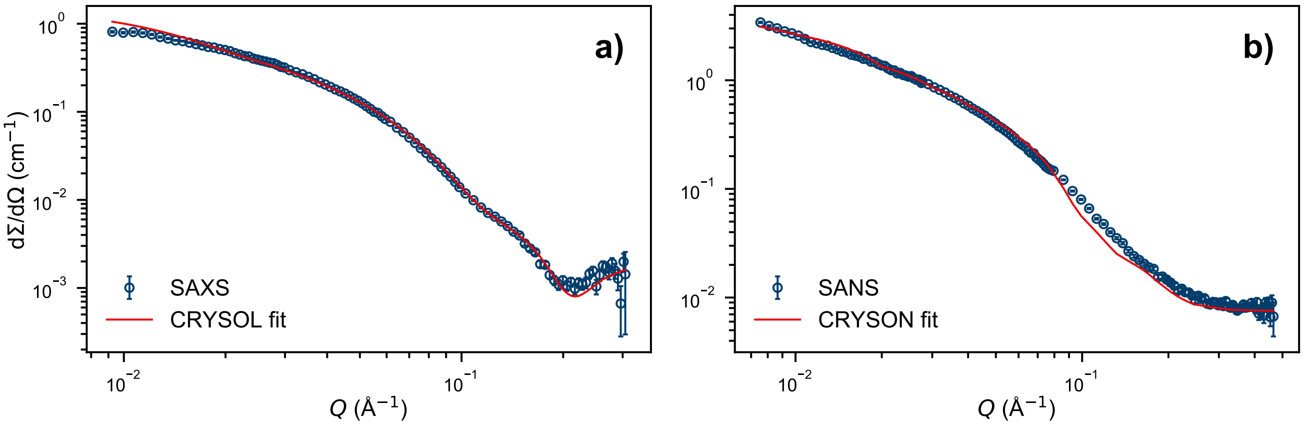

FIG. S8: The scattering data shown in Figure 3a) in the main manuscript, together with curves calculated by CRYSOL and CRYSON based on the simulated $K_3W(QL)_6K_2$ fiber structure.

SAXS DATA OF SONICATED SAMPLE

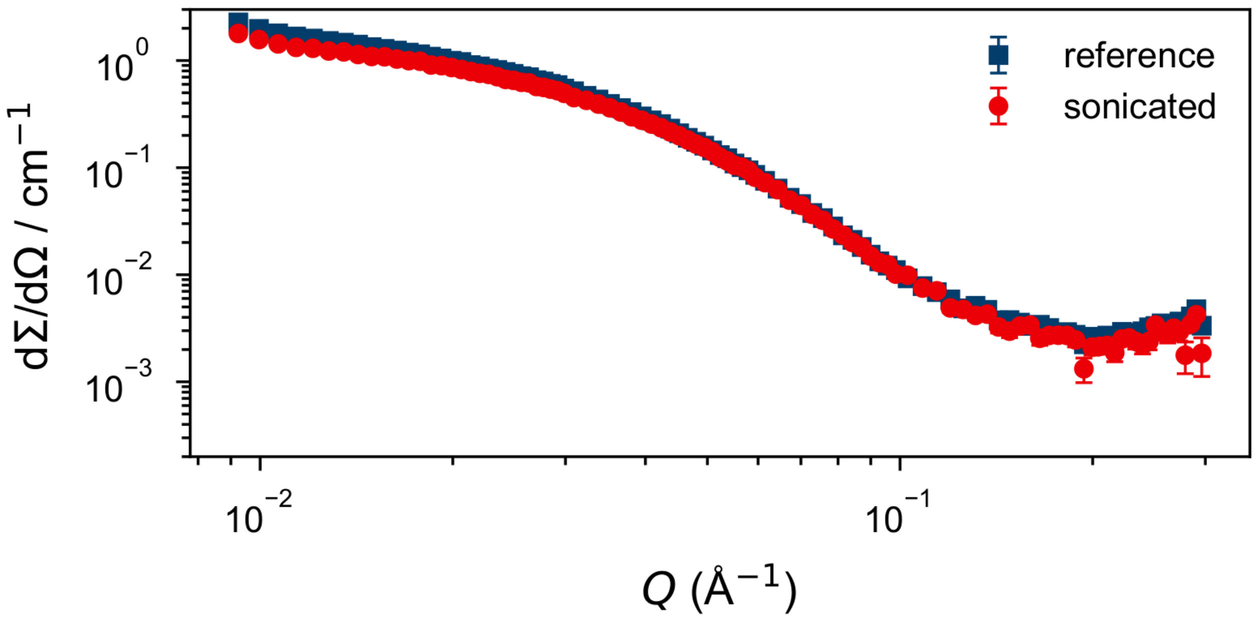

FIG. S9: SAXS data of an untreated 1:1 reference mixture of hPEG-$K_3W(QL)_6K_2$ and dPEG-$K_3W(QL)_6K_2$ in 56vol% D_2O together with the sonicated sample. Sonication did not destroy the peptide fibers. The slightly lower forward scattering may indicate somewhat shorter fibers.
